# New Cysteine-Rich Ice-Binding Protein Secreted from Antarctic Microalga, *Chloromonas* sp.

**DOI:** 10.1371/journal.pone.0154056

**Published:** 2016-04-20

**Authors:** Woongsic Jung, Robert L. Campbell, Yunho Gwak, Jong Im Kim, Peter L. Davies, EonSeon Jin

**Affiliations:** 1 Department of Life Science, Hanyang University, Seoul, South Korea; 2 Department of Biology, Chungnam National University, Daejeon, South Korea; 3 Department of Biomedical and Molecular Sciences, Queen’s University, Kingston, Canada K7L-3N6; Bose Institute, INDIA

## Abstract

Many microorganisms in Antarctica survive in the cold environment there by producing ice-binding proteins (IBPs) to control the growth of ice around them. An IBP from the Antarctic freshwater microalga, *Chloromonas* sp., was identified and characterized. The length of the *Chloromonas* sp. IBP (*ChloroIBP*) gene was 3.2 kb with 12 exons, and the molecular weight of the protein deduced from the *ChloroIBP* cDNA was 34.0 kDa. Expression of the *ChloroIBP* gene was up- and down-regulated by freezing and warming conditions, respectively. Western blot analysis revealed that native ChloroIBP was secreted into the culture medium. This protein has fifteen cysteines and is extensively disulfide bonded as shown by in-gel mobility shifts between oxidizing and reducing conditions. The open-reading frame of *ChloroIBP* was cloned and over-expressed in *Escherichia coli* to investigate the IBP’s biochemical characteristics. Recombinant ChloroIBP produced as a fusion protein with thioredoxin was purified by affinity chromatography and formed single ice crystals of a dendritic shape with a thermal hysteresis activity of 0.4±0.02°C at a concentration of 5 mg/ml. *In silico* structural modeling indicated that the three-dimensional structure of ChloroIBP was that of a right-handed β-helix. Site-directed mutagenesis of *ChloroIBP* showed that a conserved region of six parallel T-X-T motifs on the β-2 face was the ice-binding region, as predicted from the model. In addition to disulfide bonding, hydrophobic interactions between inward-pointing residues on the β-1 and β-2 faces, in the region of ice-binding motifs, were crucial to maintaining the structural conformation of ice-binding site and the ice-binding activity of ChloroIBP.

## Introduction

The Antarctic continent has one of the harshest environments on earth, with strong winds, frequent blizzards, and very low temperatures. Here, Antarctic planktonic habitats exhibit consistently low temperatures, ranging from -1.9 to 2.0 in seawater, and 0 to 5°Cin freshwater [1]. Moreover cell growth and photosynthesis of microalgae that live in ice a few meters in thickness will be affected by low solar insolation during winter and spring and, especially, by the attenuation of photosynthetically active radiation (PAR) transmitted through the snow and ice [2]. Ice formed from freshwater reduces the amount of incident irradiation that can be used by photosynthetic microalgae [3,4]. It was reported that the amount of incident irradiation decreased to <0.1% when snow covered the surface of the ice [4]. Microalgae that live just beneath or within the ice have more access to the PAR than those in the deeper water columns.

When sea ice and freshwater ice are formed, microstructures with ice-water interfaces are generated inside the ice [5]. These ice-water interfaces can form a dynamic ecosystem consisting of captured microalgae, bacteria, and dissolved organic matter that heterotrophic microorganisms are able to consume. However, in this niche, microorganisms face the danger of being surrounded and entombed by ice. As a counter to these threats, many microorganisms not capable of moving or migrating to the non-freezing locations in the polar region have developed strategies that allow for their survival [6–10]. One common adaptive strategy microorganisms use is the production and secretion of antifreeze proteins (AFPs), otherwise known as ice-binding proteins (IBPs).

In freeze—tolerant plants, IBPs are secreted into the extracellular space under cold conditions where they prevent ice recrystallization, which would otherwise lead to large ice crystals forming and doing structural damage to the tissues [11,12]. In fish and insects, IBPs are referred to as antifreeze proteins (AFPs) because they help their hosts avoid freezing. The AFPs are secreted internally into the blood or hemolymph where they bind to seed ice crystals and prevent their growth. In polar microorganisms, IBPs are secreted outside the organism to keep aqueous spaces open in the ice, which is necessary for nutrient uptake, metabolism, and the removal of waste products [13]. Thus, although many different organisms use IBPs to help their survival in sub-zero conditions, they can use them in at least three different ways.

When sea ice melts and diatoms and microalgae are allowed to settle, a clear supernatant can be obtained [14]. It was reported that the supernatant included macromolecular materials that affect the growth of ice [15,16]. The most obvious effects of the ice-active substances (IASs) are pitting and other deformities in the surface of growing ice crystals, which indicate an ability of the macromolecular materials to bind to ice [14–16]. We now recognize these IASs as a widespread type of IBP.

Numerous different types of IBP have been characterized in fish, insects, plant and microorganisms [17]. It is clear from their diverse sequences and structures that IBPs have evolved on many occasions in the different biological kingdoms. For example, there are at least four different IBP types in fishes, but two of these types have independently arisen on more than one occasion. A similar situation is seen in insects, also with remarkable examples of convergent evolution from different origins. In stark contrast, many different microorganisms, bacteria, microalgae, diatoms, yeast and fungi, have the same IBP type. The first two examples of this IBP to have their structures solved by X-ray crystallography came from an Arctic yeast and a snow mold fungus. These IBP homologues have a bipartite three-sided β-helical fold (solenoid) stabilized on one side by an α-helix that runs the length of the molecules [18,19]. This DUF3494 domain is a unique structure because of the way a single beta-helix is assembled from two distant parts of the same molecule. The only way to account for the presence of this IBP type (1^st^ IBP) in all these different microorganisms is through horizontal gene transfer (HGT). This transfer process could have been facilitated by the close proximity of microorganisms in these ice-bound niches and the huge selective advantage being able to secrete an IBP to structure the surrounding ice.

*Chlamydomonas raudensis* UW0241 produces only the 1^st^ IBP but the Antarctic *Chlamydomonas* sp. CCMP681 appears to produce a second type of IBP (2^nd^ IBP) that is unrelated to the 1^st^ IBP [20]. Recently, *Chloromonas brevipina*, which is in the same class as *Chlamydomonadaceae*, was shown to possess the 1^st^ IBP [21]. However, there is little genetic and structural information about the IBP types present in the genus *Chloromonas*.

In the current study, we found examples of the 2^nd^ IBP gene in *Chloromonas* sp. isolated from Antarctica. We have cloned and expressed the *ChloroIBP* gene and compared its gene product (ChloroIBP) with other IBPs and AFPs from various organisms. ChloroIBP is distinctly different from the other IBPs. The biochemical properties of *Chloromonas* sp. were characterized and the expression of this protein was found to be up-regulated by freezing conditions. We verified that *Chloromonas* sp. secreted ChloroIBP into the culture medium. Heterologous over-expression of ChloroIBP in *E*. *coli* confirmed that the protein binds ice, shapes ice crystals and has antifreeze activity. In addition, the protein structure of ChloroIBP was modeled in conjunction with molecular dynamics analyses. The protein folds as an extensively disulfide-bonded beta-solenoid with a triangular cross-section. One of the three faces of the solenoid is highly irregular due to many projecting peptide loops. However, another face (β2) is flat and displays an array of putative TXT ice-binding motifs.

## Results

### Antarctic microalgal strain producing a new IBP is *Chloromonas* sp.

**Fig 1:**
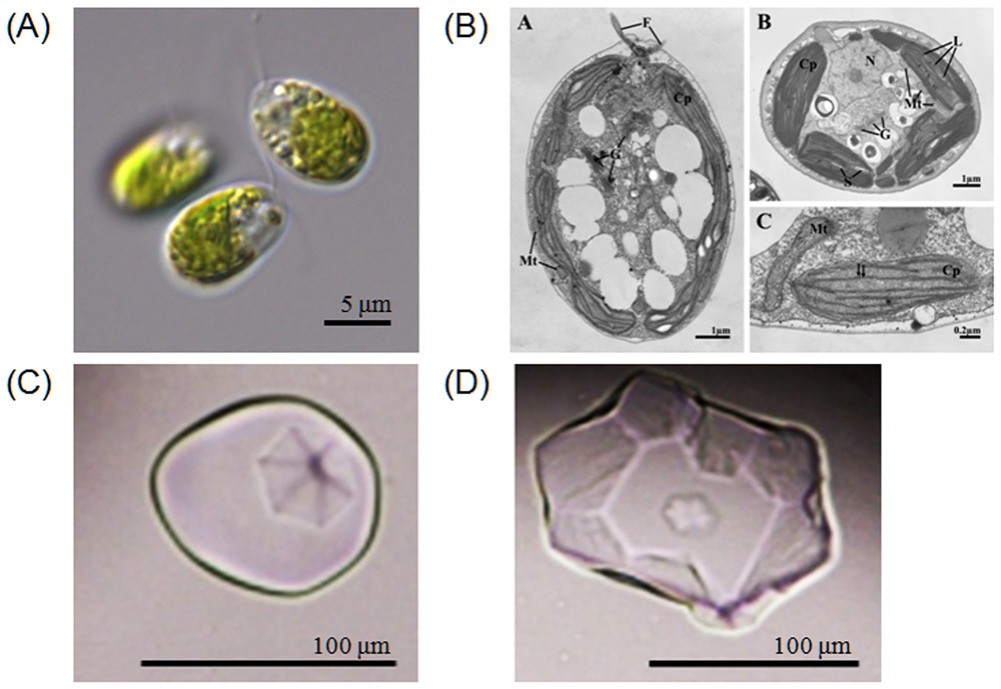
Pictures of *Chloromonas* sp., and single ice crystal shapes from the extracellular fraction of *Chloromonas* sp. (A) light micrograph showing chloroplast and flagella; (b) electron micrographs showing ultrastructural features by longitudinal and cross-section views (Cp, Chloroplast; F, Flagella; G, Golgi complex; L, Lumen; Mt, Mitochondria; N, Nucleus; S, Starch (C and D) ice crystal shapes formed by ice-binding proteins secreted from *Chloromonas* sp showing hexagonal morphology.

**Fig 2:**
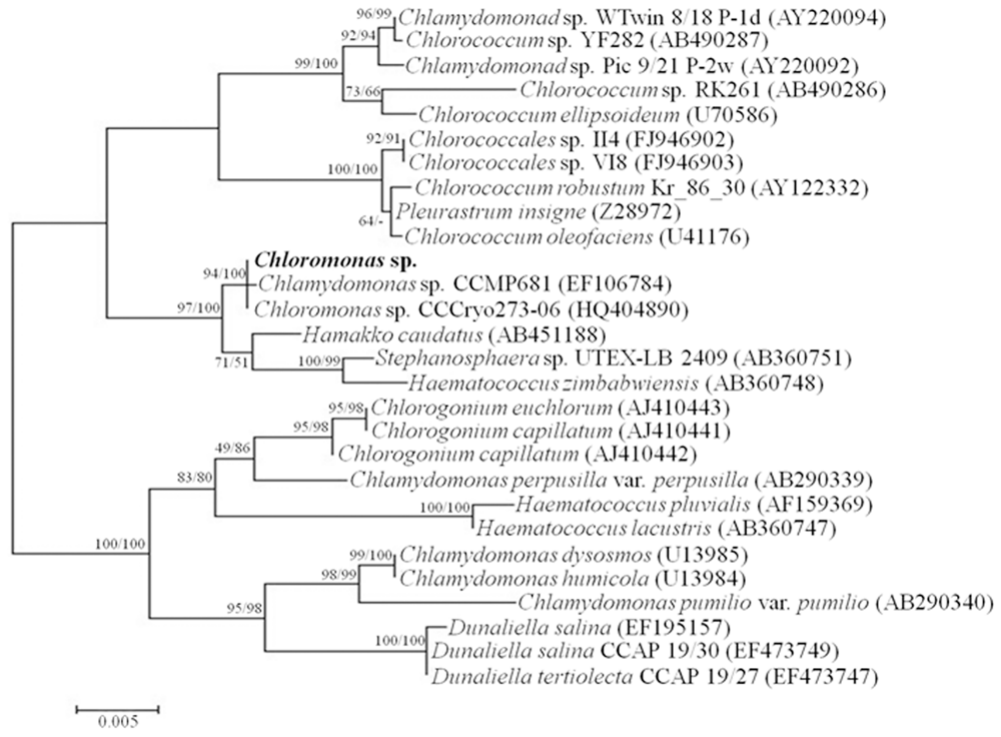
Phylogenetic tree of SSU (18S rDNA) sequences among microorganisms genetically-close to *Chloromonas* sp. The sequences used for analysis were acquired from the NCBI database and aligned by the ClustalW algorithm [24]. The tree was generated by the maximum likelihood (ML) method. The probabilities from maximum parsimony and distance methods (left and right values, respectively) were obtained by bootstrap analysis of 5000 repetitions.

**Fig 3:**
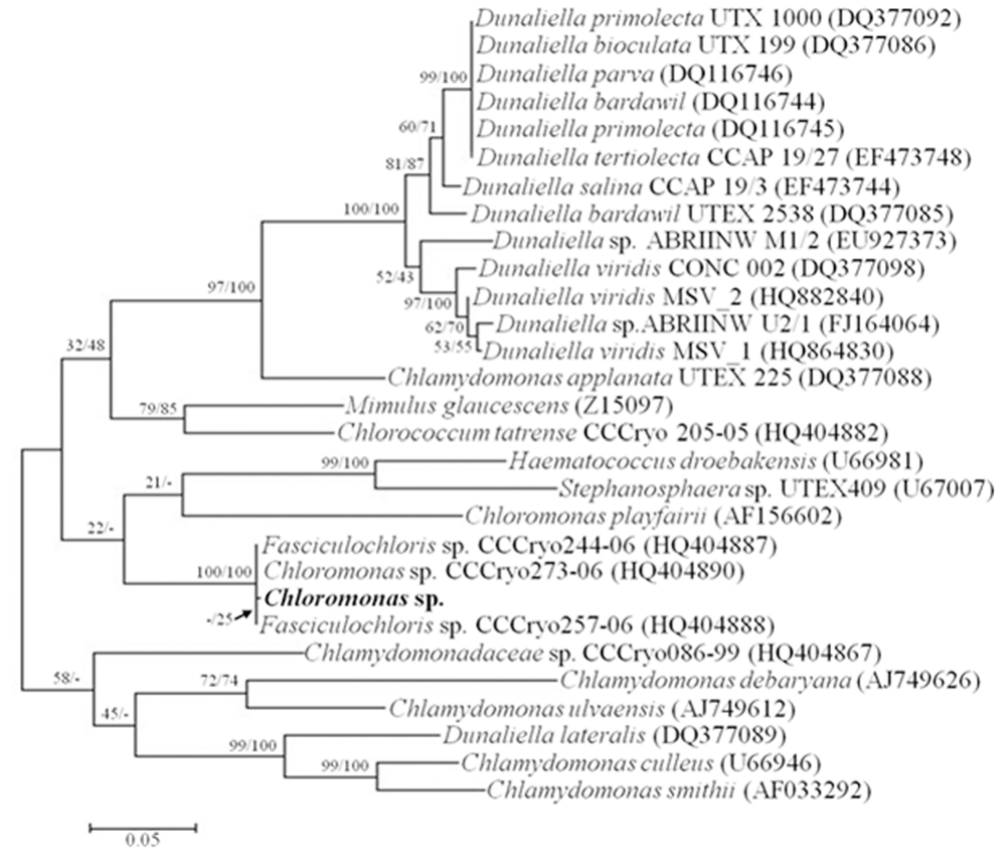
Phylogenetic tree of intergenic transcribed spacer (ITS) sequences among microorganisms genetically-close to *Chloromonas* sp. The tree was generated as described in the legend to Fig 2.

*Chloromonas* sp. was harvested near the King Sejong Station in the King George Island in Antarctica and was initially identified by optical microscopy. The *Chloromonas* sp. is unicellular with a spherical shape and a length of approximately 10 μm (Fig 1A). By using transmission electron microscopy, cellular organelles were observed more clearly, and no pyrenoid was detected (Fig 1B). To confirm the identity of the Antarctica strain, nucleotide sequences of 18S rDNA (SSU) and ITS (intergenic transcribed spacer) were analyzed. Genomic SSU (1,765 bp) and ITS (683 bp) were cloned, sequenced and aligned with the sequences in the NCBI database by nucleotide blast (megablast). The microalgal strain was identified as *Chloromonas* sp. due to having 99% maximum identity (99 and 100% query coverage of SSU and ITS, respectively) for SSU and ITS with *Chloromonas* sp. CCCryo273-06 (Genbank accession number HQ404890). The relationship between *Chloromonas* sp. and *Chloromonas* CCCryo273-06 was strongly supported by phylogenetic analyses with maximum likelihood (ML) and neighbor-joining (NJ) methods using MEGA6 (Figs 2 and 3).

### *ChloroIBP* has 12 exons

**Fig 4:**
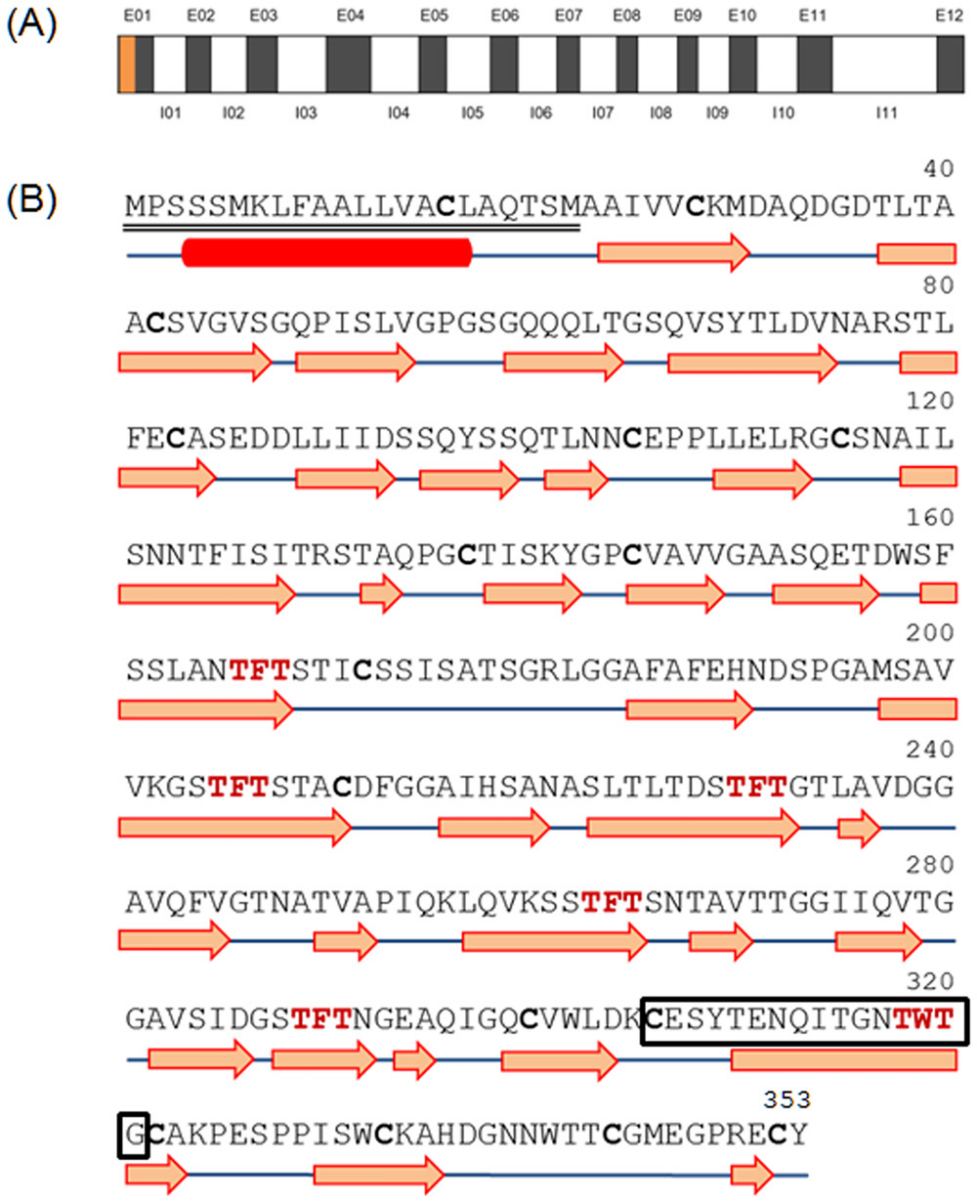
Genomic DNA structure of the ChloroIBP gene and prediction of secondary structure of ChloroIBP. (A) The genomic structure consisted of 11 introns (I) and 12 exons (E) presented by white and gray boxes, respectively. The signal peptide is coloured orange. Lengths of the introns and exons are shown in S2 Table. (B) Deduced amino acid sequence of ChloroIBP with the signal peptide sequence double-underlined. A box with black colour shows the peptide sequences for detection of ChloroIBP in western blot analysis. Potential T-X-T ice-binding motifs are displayed in a bold, red font. Cysteine residues are shown in bold letters. The red box and blue lines indicate α-helix and coils, respectively. Orange arrows indicate β-strands.

**Fig 5:**
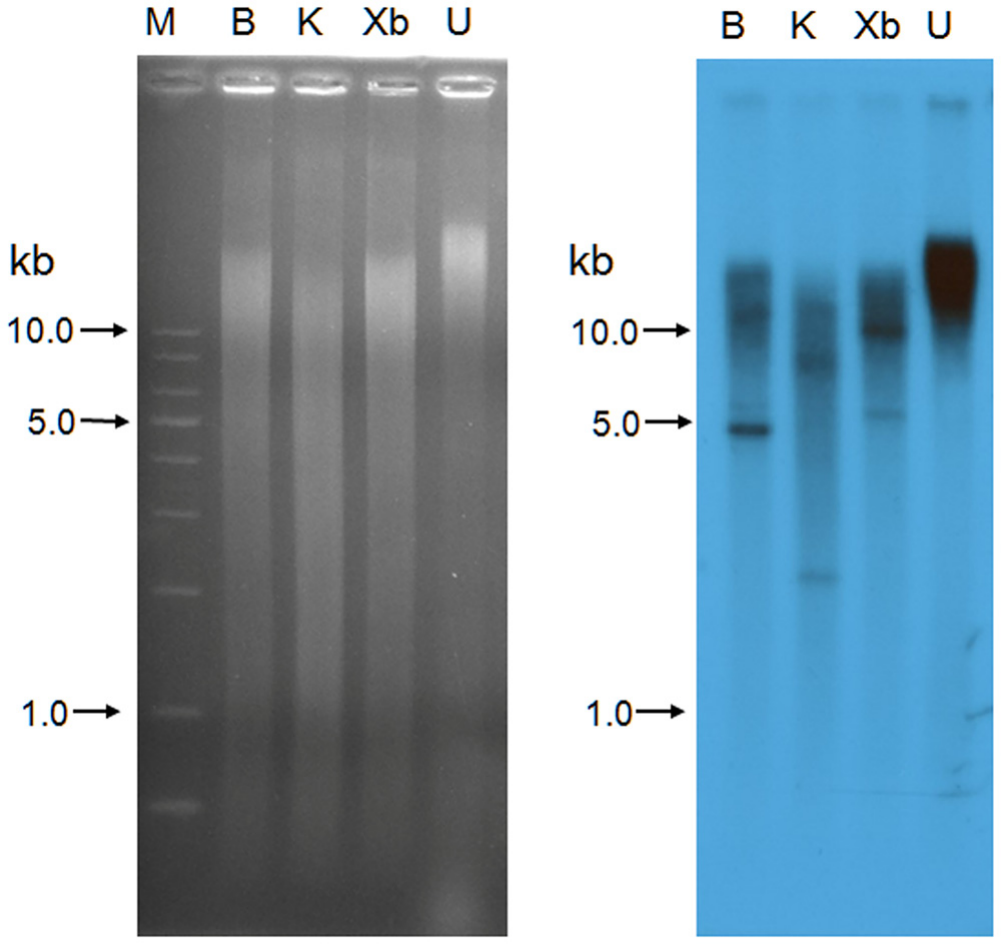
Southern blotting of Chloromonas genomic DNA to analyze the gene family encoding ChloroIBP. Left: Ethidium bromide-stained agarose gel showing: M, 1 kb DNA ladder; B, *Bam*HI-digested *Chloromonas* genomic DNA; K, *Kpn*I-digested *Chloromonas* genomic DNA; Xb, *Xba*I-digested *Chloromonas* genomic DNA; U, Undigested nuclear DNA as a control. Right: Autoradiogram of the probed blot from the gel on the left.

Polymerase chain reactions (PCR) were performed to amplify the coding regions of ice-binding proteins (IBPs) from *Chloromonas* sp. (ChloroIBP) using four sets of primers based on *Chlamydomonas* sp. CCMP681 IBP gene sequences (S1 Table) [22]. Amplified DNA bands of approximately 3.0 kb and 1.0 kb were obtained for *Chloromonas* sp. genomic DNA (CCMP681_1_gDNA) and cDNA (CCMP681_1_cDNA) from the IBP-1gene primers of *Chlamydomonas* sp. CCMP681. The genomic DNA (gDNA) and complementary DNA (cDNA) sequences of *ChloroIBP* were 3,221 bp and 1,062 bp long, respectively. A Blastn alignment showed that the cDNA of ChloroIBP (Genbank ID, KC707921) had the highest level of identity with *Chlamydomonas* sp. CCMP681 *IBP-1* (Genbank ID, EU190445; 99% of query coverage and 93% of max. identity), followed by *IBP-3* (95% of query coverage and 78% of max. identity), and *IBP-2* (85% of query coverage and 79% of max. identity). The ChloroIBP deduced from the cDNA sequence was 353 amino acids long with a predicted mass of 36.3 kDa (Table 1). A signal peptide of 23 amino acids was detected by the SignalP program [23] at the N-terminal end of the ChloroIBP. Thus, the number of amino acids and mass of the mature ChloroIBP were estimated to be 330 and 34.0 kDa, respectively (Table 1). The genomic sequence of ChloroIBP also had a high degree of similarity with the CCMP681 IBP-1 sequence (Genbank numbered, EU190441; 89% of query coverage and 94% of max. identity). The mapping of exons and introns in genomic *ChloroIBP* was performed by manually aligning the genomic DNA and cDNA sequences. The alignment showed that *ChloroIBP* was composed of eleven introns and twelve exons (Fig 4A). The copy number of *ChloroIBP* in *Chloromonas* sp. was estimated by Southern blot analysis. Two clear bands were detected for each treatment of *Bam*HI, *Kpn*I and *Xba*I restriction endonucleases (Fig 5), suggesting that *Chloromonas* sp. may have a small gene family of IBPs in its genome.

**Table 1:** Information on the ice-binding protein originating from *Chloromonas* sp.

	Premature ChloroIBP	Mature ChloroIBP
Nucleotides (bp)	1062	993
Amino acids	353	330
MW (kDa)	36.34	33.99
pI	4.47	4.40
Extinction coefficient (L mol^-1^ cm^-1^)	34950	34950

### Sequence alignment and phylogenetic analysis of ChloroIBP confirms its distinction from other IBPs

**Fig 6:**
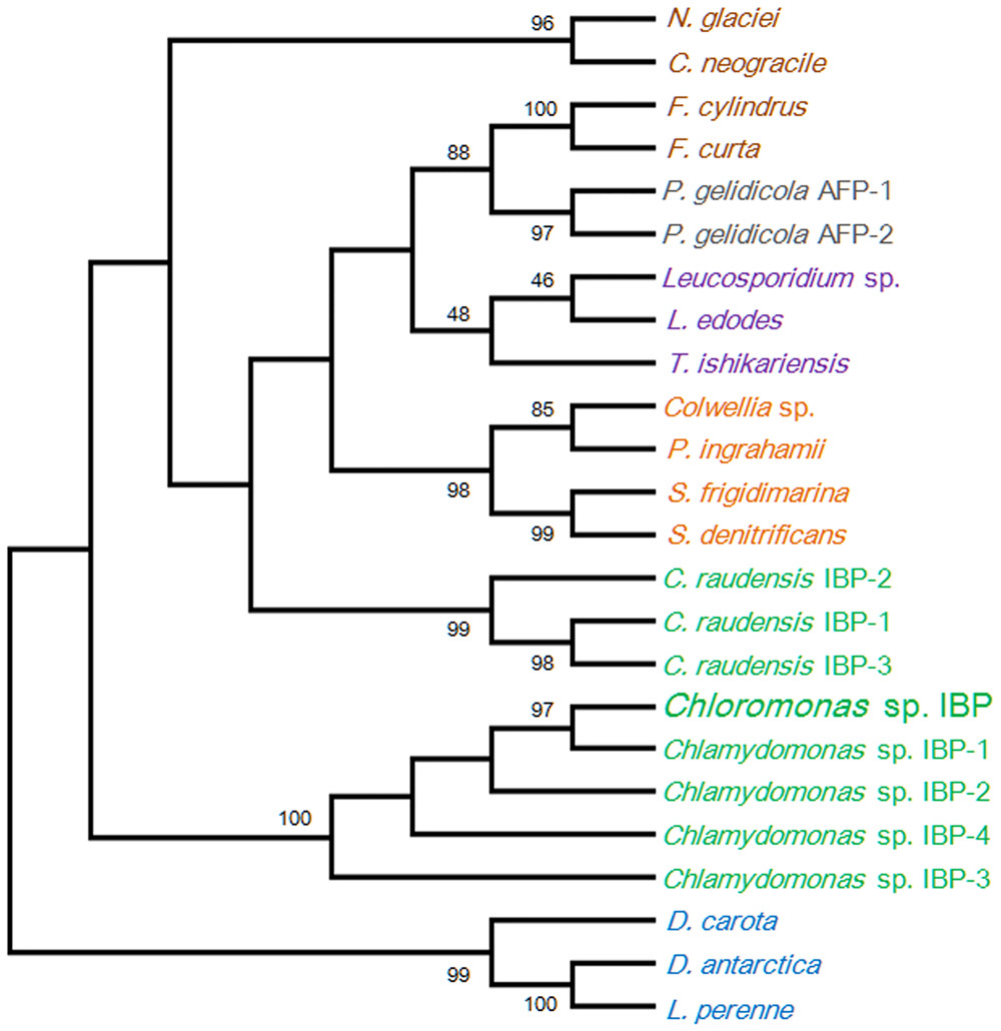
Phylogenetic relationship of ChloroIBP and various types of IBPs originating from plants and microorganisms. Amino acid sequences were aligned by the ClustalW program [24]. The phylogenetic tree was constructed by the neighbor-joining method from the MEGA 6 program [25]. The numbers in each node of the branches indicate the bootstrap values of 5,000 repetitions. Bootstrap values below 40% were rejected. Green and brown represent the Antarctic Chlorophyta and Bacillariophyceae, respectively. Orange and violet indicate bacteria and fungi, respectively. Blue represents the Planta, while grey indicates Prasinophyta.

The amino acid sequence of ChloroIBP (Genbank ID, AHF22079) was highly similar to those of four IBP isoforms produced from *Chlamydomonas* sp. CCMP681 (CCMP681), which have not yet been structurally described (S2 Fig). A comparison of the amino acid sequences by the PSI-BLAST algorithm showed that IBP-1 of *Chlamydomonas* sp. CCMP681 had the highest similarity to ChloroIBP (Genbank ID, ABY64762; 99% of query coverage and 90% of max. identity), followed by IBP-2 (97% of query coverage and 75% of max. identity), IBP-3 (96% of query coverage and 76% of max. identity) and IBP-4 (98% of query coverage and 49% of max. identity). To investigate the relationship of IBPs and AFPs originating from various organisms, a sequence alignment was performed by the ClustalW2 program [24]. Phylogenetic analysis was carried out by the MEGA6 program [25] and the phylogenetic tree was verified by the bootstrap analyses by 5,000 replications of distance method. The findings strongly supported a relationship between *ChloroIBP* and IBP-1 of *Chlamydomonas* sp. CCMP681 (with over 90% of bootstrap values) and the other IBP *Chlamydomonas* isoforms of having the same phylogenetic clade distinct from other organisms. The green microalgal IBP clade was moderately related to the plant IBP, *Secale cereale* IBP, with 44% probability, and formed genetic groups isolated from the IBPs originating from bacteria, fungi and diatoms (Fig 6).

### *ChloroIBP* expression is upregulated under freezing conditions

**Fig 7:**
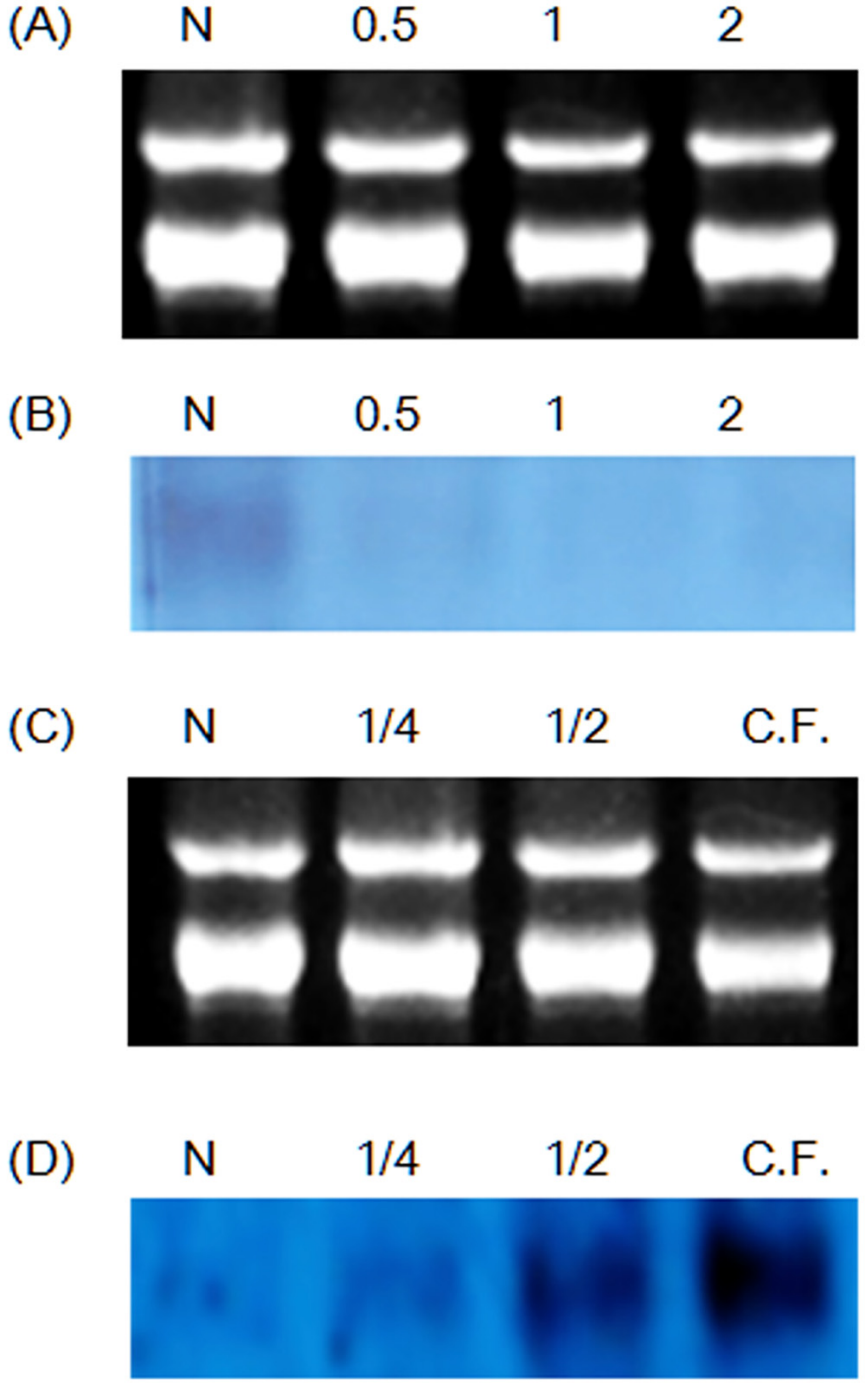
Northern blot analysis of thermal and freezing stresses. (A and C) Electrophoretic data for control RNAs. (B) Autoradiogram of transcriptional change in ChloroIBP mRNA levels with thermal conditions. N, normal cells; 0.5, 30-min incubation at 25°C; 1, 1 h-incubation at 25°C; 2, 2-h incubation at 25°C. (D) Autoradiogram of transcriptional change in ChloroIBP mRNA levels with freezing condition. N, normal cells; 1/4, 25% of medium occupied by ice slush; 1/2, 50% of medium occupied by ice slush; C.F., 100% of medium occupied by ice slush.

**Fig 8:**
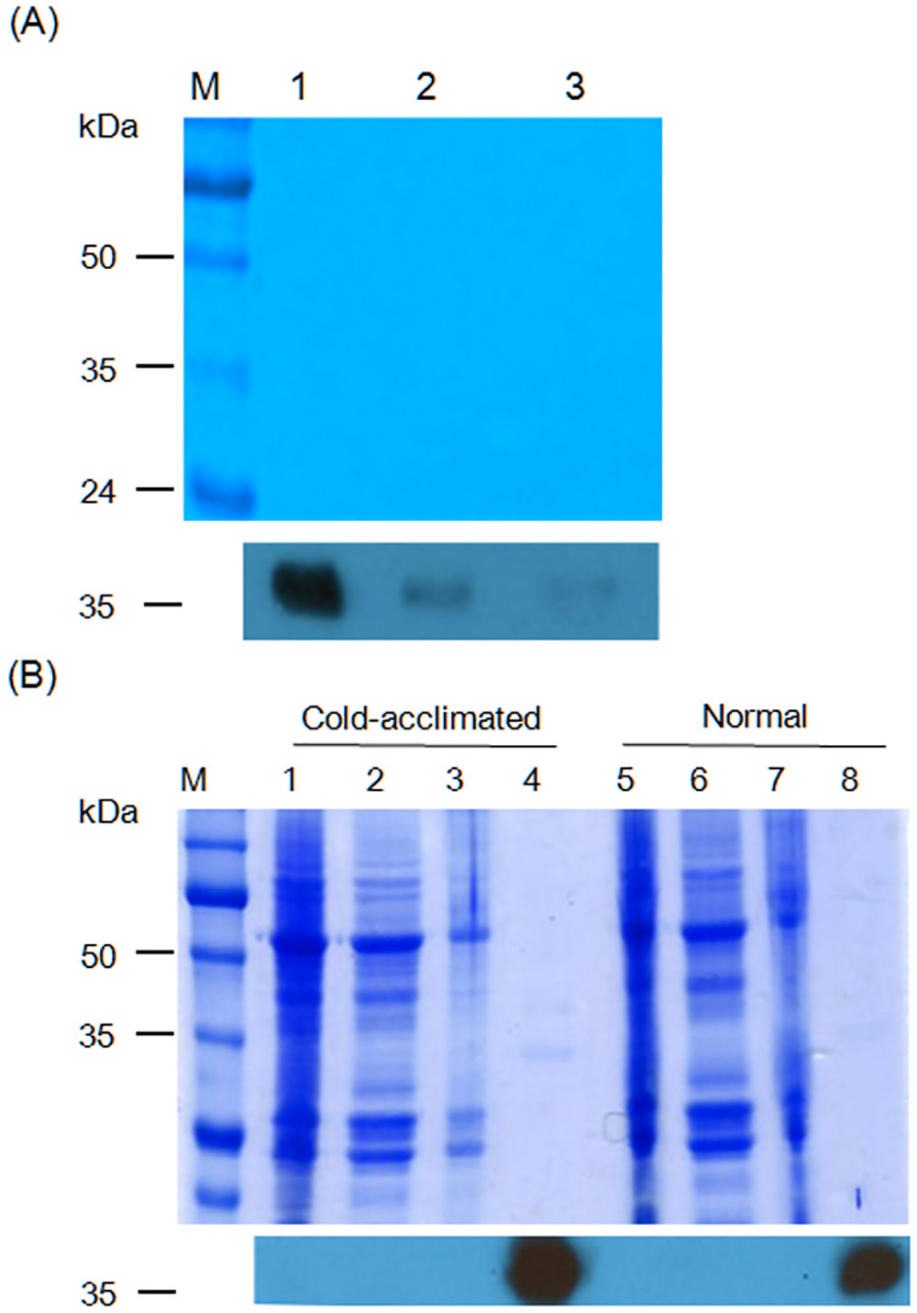
Localization and levels of ChloroIBP production according to freezing condition. (A) Top—SDS-PAGE analysis of extracellular proteins from frozen cultures (1); culture where one quarter of the volume is ice slush (2); and culture grown under normal conditions (3). Bottom—immunoblot of the gel transfer where ChoroIBP migrates. (B) SDS-PAGE and immunoblot analysis. Top—Coomassie blue staining of samples prepared at the cold-acclimated (0°C, shaking incubation for 3 days, lanes 1–4) and normal (4°C, lanes 5–8) conditions. M, Marker proteins; 1 and 5, Total crude extracts; 2 and 6, Intracellular soluble proteins; 3 and 7, Cell debris samples; 4 and 8, Extracellular proteins. Bottom—immunoblot of the gel transfer where ChoroIBP migrates.

In order to investigate the response of *Chloromonas* sp. to environmental stress, the effect of change of temperature on the ChloroIBP expression was analyzed at the transcriptional and translational level by northern and western blot analyses, respectively. Incubation of *Chloromonas* sp cultures for 30 min, 1 h and 2 h at 25°C (thermal stress) clearly reduced the ChloroIBP transcript levels (Fig 7B). In contrast, *Chloromonas* sp. IBP transcript levels increased with the extent of freezing of the cultures (Fig 7D). Transcript levels were increased several fold when ice formed to one quarter of the culture volume in comparison to levels in the normal culture conditions at 4°C. The transcript levels were further increased when one half of the culture froze, and again when the entire culture appeared frozen. To see if the amount of ChloroIBP produced matched these transcript levels; the former was analyzed by western blotting (Fig 8A). *Chloromonas* sp. expressed IBP under control conditions, as evidenced by ice crystal shaping in the culture media (Fig 1C and 1D). When the ChloroIBP signals from the immunoblot were revealed, they were many times more intense in samples exposed to ice (Fig 8A, lanes 1 and 2) than those in ice-free culture (lane 3). Thus, production of IBP in *Chloromonas* sp. is especially increased under the conditions of subzero temperatures when the cells encounter a freezing environment.

### ChloroIBP is secreted into the external environment

ChloroIBP has an N-terminal signal sequence and should be directed outside the cell during synthesis. To confirm the location of ChloroIBP, western blot analysis using a ChloroIBP-specific polyclonal antibody was performed. Samples were incubated at 0°C with shaking and under continuous light for one day. In *Chloromonas* sp. incubated in both normal and cold-acclimated conditions, a clear IBP protein band was detected only in the extracellular fraction (filtered concentrates of the culture medium). The ChloroIBP expression increased in the cold-acclimated condition compared to that of the normal condition, as shown by immunoblot analysis (Fig 8B). No IBP protein band was detected in the protein-rich total crude cell lysate, cytosolic fraction (intracellular proteins), and cell debris after cell lysis. These results showed that ChloroIBP was secreted into the medium (Fig 8B, lanes 4 and 8) where a single faint band of proteins at 35 kDa corresponded with the antibody signal. The diagnostic hexagonal shaping of ice crystals that indicated the presence of an IBP was observed only in the extracellular fractions (Fig 1C and 1D. These results clearly support the notion that ChloroIBP is secreted into the extracellular medium where it can inhibit the recrystallization and growth of ice crystals that might pose a danger to the alga.

### Native ChloroIBP is post-translationally modified

**Fig 9:**
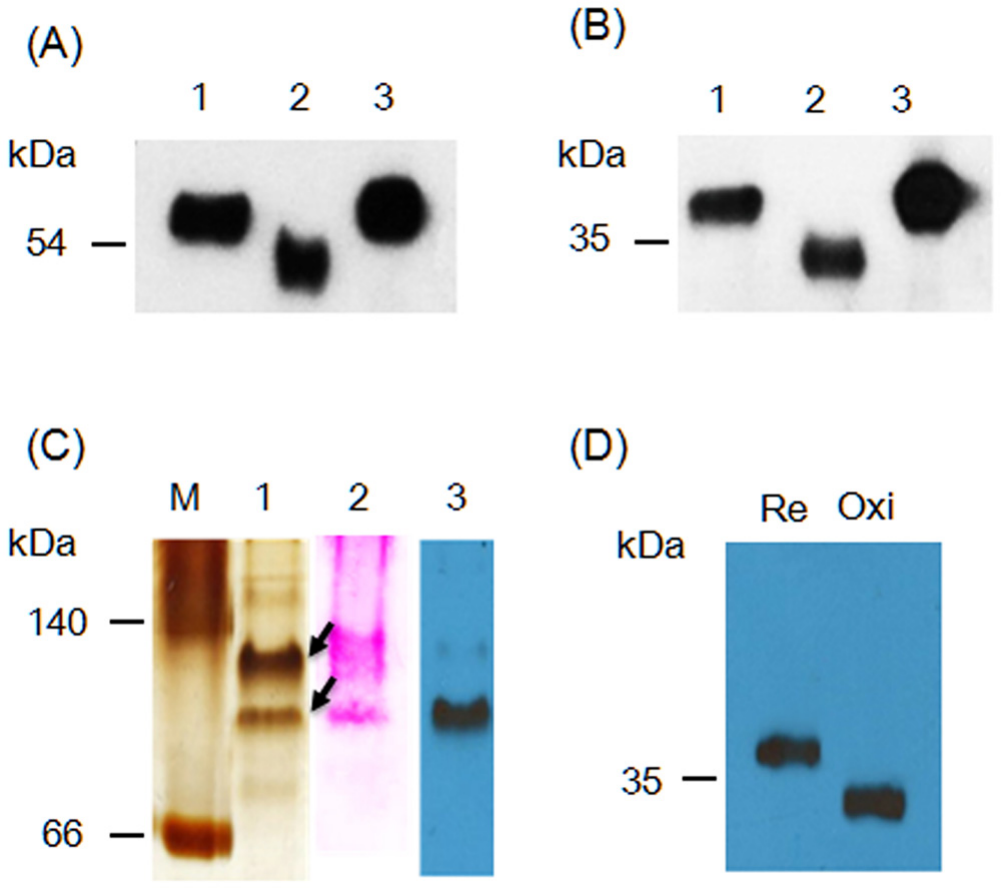
In-gel mobility of ChloroIBP. (A) Extracellular recombinant Trx-ChloroIBP visualized by immunoblotting following SDS-PAGE analysis in a redox experiment. 1, Trx-ChloroIBP treated with β-mercaptoethanol; 2, Trx-ChloroIBP oxidized by ambient air; 3, Trx-ChloroIBP alkylated by iodoacetamide after treatment with β-mercaptoethanol. (B) Topological change of native ChloroIBP. Treatment with reagents was the same as for (A). (C) Location of native ChloroIBP secreted into culture media on a native polyacrylamide gel. (M, protein markers representing bovine serum albumin based on the protein structure of P.69 pertactin (66) and L-lactic dehydrogenase (140); 1, silver staining of extracellular proteins secreted from *Chloromonas* sp.; 2, Periodic-acid staining of extracellular proteins from *Chloromonas* sp.; 3, Immunoblot band detected by anti-ChloroIBP antibody to a blot of Lane 1. (D) Topological movement of native ChloroIBP separated on a native polyacrylamide gel after reduction (Re) or under oxidizing conditions (Oxi).

Disulfide bond formation and glycosylation of the ChloroIBP were investigated by in-gel mobility shift analysis. ChloroIBP has fifteen Cys residues in its mature sequence (S1 Fig). The theoretical molecular weights of recombinant Trx-ChloroIBP and native Chloro IBP are 51.4 and 34.0 kDa, respectively, as computed by the pI/Mw program in ExPASy package [26]. The 55 kDa and 36 kDa values obtained by SDS-PAGE after reduction by β-mercaptoethanol are within 2–4 kDa of these values (Lane 1 in Fig 9A and 9B). Under oxidizing conditions these bands were shifted to lower apparent molecular weights of 51 and 34 kDa for Trx-ChloroIBP and native ChloroIBP, respectively (Lane 2 in Fig 9A and 9B). The downward shift to lower molecular weights is consistent with disulfide bond formation between Cys residues producing more compact forms of ChloroIBPs with higher mobility in the gel system.

Two putative N-glycosylation sites were predicted at 122N and 248N by the NetGlyc program (S3 Fig) [27]. To determine if native ChloroIBP is glycosylated, the secreted protein from *Chloromonas* sp. was loaded on a native polyacrylamide gel and transferred to a nylon membrane to perform periodic-acid staining. Two major protein bands were detected on the native gel by silver-staining (Fig 9C, lane 1) running faster than the 140 kDa marker. The same two bands were detected on a PVDF membrane by a periodic-acid staining after western transfer (Fig 9C, lane 2). Immunoblot analysis with the native extracellular proteins of *Chloromonas* sp. showed that only the lower band reacted with anti-ChloroIBP antibodies. No bands were detected below the 66 kDa marker on either the silver-stained native gel or the western blot result. The lower band of native ChloroIBP exhibited the same pattern of conformational change of the native ChloroIBP under reducing and nonreducing conditions in the SDS-PAGE (Fig 9D). Therefore, native ChloroIBP secreted from *Chloromonas* sp. cells appears to be both disulfide bonded and glycosylated.

### Recombinant ChloroIBP behaves as a weak antifreeze protein

To adequately characterize individual IBPs it is necessary to produce them as properly folded recombinant proteins [18,19,28,29]. Given that ChloroIBP might have as many as 7 disulfide bonds, we elected to produce it as a fusion protein with thioredoxin to help both solubility and disulfide bond formation in the cytoplasm of *E*. *coli* [30–32]. When ChloroIBP fused with the TrxA domain (Trx-ChloroIBP) was expressed in *E*. *coli*, a large amount of Trx-ChloroIBP appeared in the soluble fraction as a thick band at the molecular weight of approximately 54 kDa, which was the expected size of the fusion protein. (S4 Fig). TrxA-ChloroIBP was purified from soluble fractions by Ni-NTA affinity chromatography (Lane 5 in S4 Fig).

**Fig 10:**
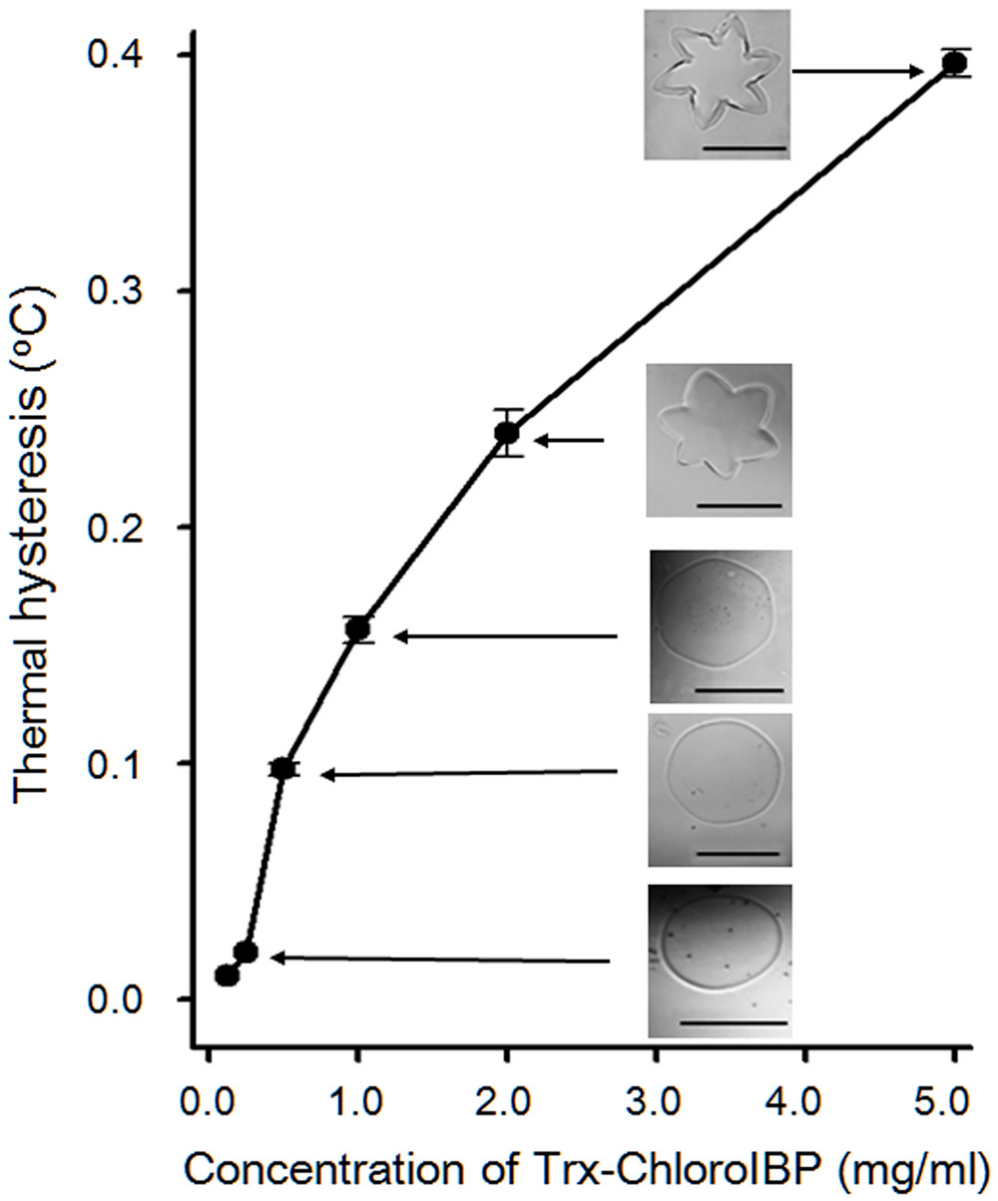
Thermal hysteresis activity as a function of Trx-ChloroIBP concentration. Insets show the morphological changes of single ice crystals at different recombinant ChloroIBP concentrations. BSA solution (5 mg/ml) was used as a control. Scale bars indicate 100 μm.

Purified Trx-ChloroIBP was concentrated to 5 mg/ml and tested for thermal hysteresis (TH) activity and ice crystal shaping. At this concentration Trx-ChloroIBP changed the single ice crystal shape from a smooth disc to a symmetrical star-shaped morphology, and lowered the freezing temperature below the ice melting point (thermal hysteresis) by 0.4±0.02°C (Fig 10). TH activity decreased with dilution of the stock IBP solution and was not detected below a concentration of 0.25 mg/ml. Based on the comparative analysis of antifreeze activity between Trx-ChloroIBP and that of other IBPs and AFPs, Trx-ChloroIBP was shown to have an antifreeze activity quantitatively similar to that of microalgal and plant IBPs, and shaped ice into a star-like single ice crystal morphology that is also seen with the wild-type ChloroIBP.

### Modeling and molecular dynamics of ChloroIBP suggest it folds as a beta-solenoid structure

Three-dimensional analyses of IBPs have been performed to better understand the relationship between protein structure and an IBP’s ability to bind to, and stop the growth of ice [33–38]. As a prelude and aid to the structural biology approach it is worthwhile to attempt a prediction of the target protein’s structure, *in silico* protein modeling and molecular dynamics were used. The deduced amino acid sequence of ChloroIBP was entered into the Phyre2 server [39]. By pairwise alignment based on the BLOSUM62 algorithm ChloroIBP had a sequence identity of 12.2% with P.69 pertactin, a virulence factor from *Bordetella pertussis* (PDB ID, 1DAB). The match of ChloroIBP to P.69 pertactin was particularly convincing in the N-terminal region. Homology modeling was performed to fit the protein structure of ChloroIBP to P.69 pertactin using reiterative analysis by the Modeller program [40].

**Fig 11:**
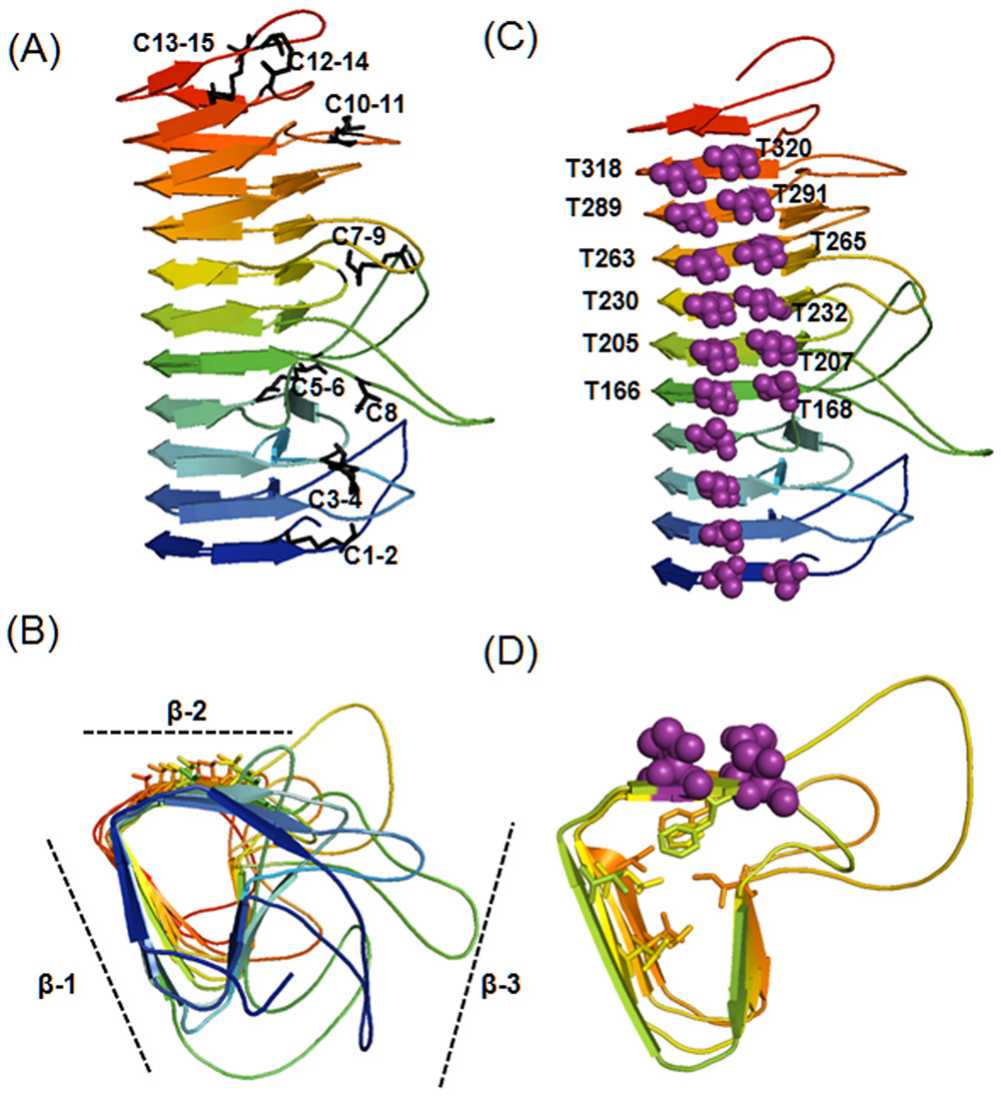
Overall *in silico* three dimensional structure of ChloroIBP from *Bordetella pertussis* (1DAB). Homology modeling was performed by the Modeller v.9.9 program [40]. The results of protein modeling were visualized by the program PyMol v1.3 [79]. (A) Rainbow colours blue to red mark the progression along the protein backbone from N to C termini, respectively. Possible formation of disulfide bonds was predicted by the DIpro program [44] in the SCRATCH protein predictor. Residue numbers of the Cys are indicated as follows. C1, Cys28; C2, Cys42; C3, Cys83; C4, Cys105 C5, Cys115; C6, Cys137; C7, Cys145; C8, Cys172; C9, Cys211; C10, Cys300; C11, Cys306; C12, Cys322; C13, Cys333; C14, Cys344; C15, Cys352. C8 (denoted by an asterisk) was assumed to be a free Cys residue retaining a free thiol group. All other Cys are paired according to the modeling prediction. (B) The end-on view of ChloroIBP is displayed with the same colour scheme as in (A). ChloroIBP showed three β-faces including the β-2 face analyzed to have the T-X-T motifs indicated by sticks. (C) The ChloroIBP β-2 face showing the disposition of Thr residues (purple spheres). The numbers of Thr in T-X-T motifs substituted with Tyr in site-directed mutagenesis are indicated along the corresponding residues. (D) The hydrophobic core of ChloroIBP composed of Val, Leu, Ile, Phe, and Trp as indicated by the stick representation of their side chains.

Alignment and superimposition of the amino acid sequences of ChloroIBP and the corresponding regions of P.69 pertactin suggested that ChloroIBP forms a beta-solenoid structure with eleven three-sided coils [41]. Two sides inclined at ~ 70° to each other are formed from remarkably flat, regular beta-sheets (Fig 11A and 11B) [41]. The distance between each coil of the β-solenoid was estimated to be ~4.8 Å, on average. The third side of the structure was concave, which served to reduce the cross-sectional area of the core and eliminate any cavities that might destabilize the fold. This third side was also interrupted by multiple loops which had no equivalents in pertactin and could not be accurately forecast in the overall structure. Deconvolution of the averaged CD spectra for the 495-residue Trx-ChloroIBP showed a third of the protein was beta-structure and one third was coil, whereas turns and α-helix comprised 20% and 13% of the structure, respectively (S6 Fig and S3 Table). In the analysis of root-mean-square (RMS) deviation and radius of gyration, the structures in the trajectory were fit using only the alpha carbons of the core residues (S7A and S7B Fig). This showed that the core was quite stable while the loops moved quite a bit from their starting positions. This result was also evident in the RMS fluctuation plot that showed the average movement of the alpha carbons of the loop residues (S7C Fig). The radius of gyration plot used all of the atoms of the protein and also showed some fluctuation that could be attributed to the loop movements. (S7B Fig). The loops all undergo some significant movements, some of which result in the collapse to a more compact structure. This division of secondary structure for the whole fusion protein is a good match to the model of the IBP portion (Fig 11), which makes up three quarters of the mass of Trx-ChloroIBP. The only discrepancy was in α-helix content, which is minimal in the model. In fact, much of the α-helix content comes from the one quarter of the structure that is thioredoxin, where it is the most abundant secondary structure (31%). Once the thioredoxin contribution to secondary structure was subtracted, the helix content fell to 7% and the amount of beta-structure and coil increased slightly.

Additional structural information can come from the presence of disulfide bonds linking different regions of ChloroIBP in three-dimensional. To localize the 15 Cys residues in mature ChloroIBP and predict the disulfide bond partners, analyses were conducted using PeptideCutter [42] and PeptideMass [42,43] in ExPASy, and DIpro [44] in the SCRATCH protein predictor programs. From the series of analyses used to identify the potential disulfide bonds, it was predicted that ChloroIBP has seven potential disulfide bonds making intramolecular links between Cys 1 and 2, Cys 3 and 4, Cys 5 and 6, Cys 7 and 9, Cys 10 and 11, Cys 12 and 14 and Cys 13 and 15 (Fig 11A). The free cysteine is predicted to be Cys 8. All 15 Cys are clustered on the concave site of the solenoid and appear to cross-link the loops. There is a tight hydrophobic core between the flat beta-sheets that includes a series of six pi-stacked aromatic residues (5 Phe and 1 Trp) contributed by neighbouring coils.

Having established that the three-dimensional structure of ChloroIBP is a *β*-solenoid with a right-handed coiling and a left-handed twist (S5A Fig), we next looked for possible ice-binding residues/motifs. Within the linear sequence of ChloroIBP there are six Thr-X-Thr motifs (or variations thereof), where X- is an inward pointing residue. This is a common ice-binding motif in beta-solenoid IBPs, where the Thr form two outward-pointing parallel arrays that can order water molecules into an ice-like pattern (S5B and S5C Fig).

Mutual support for both the three-dimensional ChloroIBP model and the involvement of Thr-X-Thr in ice-binding comes from the localization of the six ice-binding motifs in a parallel array on one of the two flat beta-sheets (the β-2 face). In this instance X of the motif is an inward-pointing aromatic residue (Phe or Trp) that stacks together in the hydrophobic core. (Fig 11D). This topology ensures that all six putative ice-binding motifs are positioned on the β-2 face in a regular array. The model also predicts a string of four more Thr extending one rank of the Thr-X-Thr motifs to the N terminus of the protein.

### Site-directed mutagenesis of recombinant ChloroIBP supports the model and identifies the threonine-rich surface as the ice-binding site

**Fig 12:**
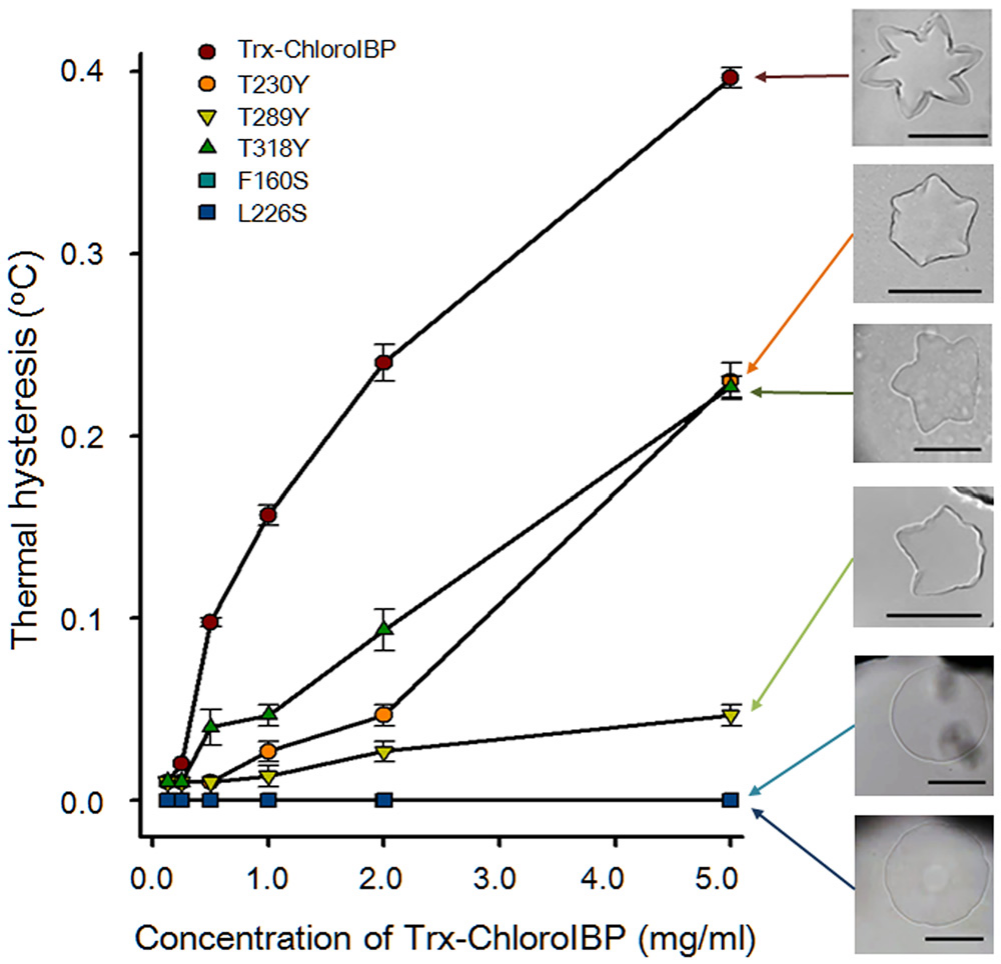
Plot of thermal hysteresis activity as a function of protein concentration of wild-type and mutant Trx-ChloroIBP. Colours and symbols indicate the different ChloroIBP mutants. Values shown are averaged S.D. of three replicates of the measurement of TH. Insets show single ice crystal morphology obtained with each Trx-ChloroIBP at the highest protein concentration assayed. Scale bars indicate 100 μm.

The interactions between hydrophobic residues in the hydrophobic core of the β-solenoidal protein structure are crucial for sustaining the β-helical structure [41]. To verify the accuracy of the three-dimensional model, predicted hydrophobic interactions in the core of ChoroIBP were probed by two mutations in which Phe and Leu were substituted with Ser (F160S and L226S). The activities these two mutant proteins, where hydrophobic residues were replaced with much smaller hydrophilic serine, were completely eliminated and the single ice crystals formed by both mutants had a circular disk shape (Fig 12 and S8 Fig).

**Fig 13:**
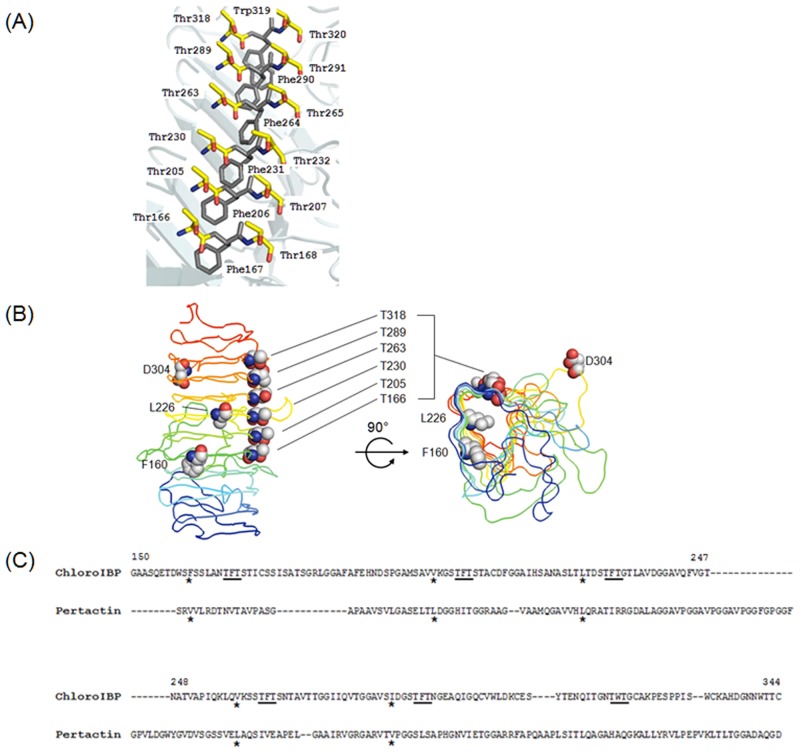
Regularity of ice-binding motifs (IBMs) on the β-2 face of ChloroIBP. (A) Six ice-binding IBMs of ChloroIBP. T-F-T and T-W-T as IBMs were illustrated by the PyMol program [79]. Red and blue indicate O and N atoms, respectively. (B) Location of some residues targeted for mutagenesis including Thr on the β-2 face. These hydrophobic residues were located closely in the hydrophobic core. (C) Alignment of amino acid sequences between ChloroIBP and pertactin P.69 in one section of the IBM region of ChloroIBP. IBMs are indicated by underlining. Hydrophobic residues predicted to interact with Phe residues in IBMs are indicated by asterisks.

To test if the ice-binding activity of ChloroIBP resides in the repetitive Thr-X-Thr residues several site-directed mutations were made on the β-2 face by mutating the second Thr to Tyr (Fig 13A and 13B). T166Y, T205Y, T230Y, T263Y, T289Y, and T318Y single mutants were each produced in the *E*. *coli* expression system. All these T to Y mutants showed substantially decreased antifreeze activity (Fig 12 and not shown). Among the six T-X-Y mutants, T289Y showed the lowest activity at approximately 10% the activity of wild-type ChloroIBP at the concentration of 5 mg/ml. In contrast to the morphology of the star-like single ice crystal from the wild-type ChloroIBP, single ice crystals of mutated ChloroIBPs formed an irregular star-like or hexagonal shape at the concentration of 5 mg/ml (S8 Fig). Based on the results of TH measurement and observation of the corresponding morphology of single ice crystals, it appears that the ice-binding site is indeed the Thr-rich beta-sheet (β2).

## Discussion

Bioinformatics analyses of ChloroIBP and its *Chlamydomonas* orthologues make it clear that this is a new type of IBP, which is completely distinct from all other IBPs currently known in fish, insects, plants and microorganisms [21,45]. It is somewhat surprising that these two distantly related algae should have the same IBP type when another *Chlamydomonas* species (*raudensis*) has the DUF3494 type of IBP as does another *Chloromonas* species (*brevispina*). There are two possible explanations for this occurrence. One is that either the *Chloromonas* sp. studied by Raymond [21] or the *Chloromonas* sp. described here has been misidentified and belongs to the other genus. The other is that the ChloroIBP gene has been horizontally transferred between these species long after they diverged. This is a plausible explanation based on the remarkable spread of the DUF3494 domain between microorganisms [21]. This single highly distinctive IBP domain is common in psychrophilic bacteria and has apparently been spread from there to algae, diatoms, yeast, fungi and even copepods [21,45].

*Chloromonas* sp. is widely distributed in freshwater and snow-covered continental regions, including Antarctica [22,46–48]. According to previous reports, the freshwater strain isolated in Antarctic temporary ponds is *Chloromonas* sp. [48,49]. *Chloromonas* sp. in this study was identified using molecular biological methods and optical and transmission electron microscopy (TEM) to verify the authenticity of the strain. Conventionally, the genus *Chloromonas* is present in volvocalean microalgae and can be distinguished morphologically from the genus *Chlamydomonas* because *Chloromonas* lacks pyrenoids in its chloroplast [49–51]. No pyrenoids were observed in the Antarctic *Chloromonas* sp. used in this study, as evidenced by TEM observation. Furthermore, this green microalgal strain belongs to the genus *Chloromonas* based on the phylogenetic analysis of DNA sequences from both 18S rDNA and intergenic transcribed spacer (ITS). Note that *Chloromonas brevispina* was taken from Lake Boney (77°43´S, 162°22´E) [21] and *Chloromonas* sp. was harvested from King George Island (62°01´S58°21´W) where the King Sejong Station is located. These two places are far apart and their ecological niches and climates are also different.

Recently, it was proposed that IBP genes might have been spread from Antarctic sea ice microorganisms by horizontal gene transfer (HGT) [20,45]. HGT is suggested to have occurred between microorganisms such as bacteria, diatoms, and green microalgae [52–54]. For Antarctic sea-ice diatoms, their IBP genes could have been transferred from ice-associated prokaryotes by HGT, which is consistent with the high identity of their amino acid sequences and the lack of introns in the microalgal IBP genes [45]. Moreover, HGT of IBP genes to Antarctic *Chlamydomonas* species has been proposed previously [20]. It was proposed that the DUF3494 IBP of *Chloromonas brevispina* appeared by horizontal gene transfer (HGT) from bacteria because all the IBPs of snow ice bacteria and fungi included the DUF3494 domain [21]. HGT of the IBP might have occurred from *Chaetoceros neogracile* to *Stephos longipeds* and from the basidiomycete group (*Flammulina populicola*, *Lentinula edodes*, *Typhula ishikariensis*, and *Leucosporidium* sp.) to the ancestral lineage of the *Fragilariopsis* clade [55,56]. What may have facilitated the gene transfer is that these organisms would be in intimate contact in fissures, channels and cavities bounded by ice where the production of ice controlling proteins to keep liquid water present would be highly selected for. The gene for the ice-binding protein from *Chloromonas* sp. (*ChloroIBP*) was identified by comparison to the four ice-binding protein isoforms in the Antarctic *Chlamydomonas*-like strain (CCMP681). This gene does not encode a DUF3494 domain. A single gene with high similarity to the *IBP-1* gene of CCMP681 was amplified from *Chloromonas* sp. Even though *ChloroIBP* showed high identity to *Chlamydomonas IBP-1* in nucleotide sequence it has one more intron and exon compared to the genomic DNA of *IBP-1* (Fig 4 and S2 Table). Given the intron-exon structure of the ChloroIBP gene it seems unlikely that this has arisen in Chloromonas as a result of HGT from bacteria.

The virulent bacterium *Bordetella pertussis* virulence factor P.69 pertactin was used for the initial three-dimensional modeling of ChloroIBP. There are several β-helix repeats in its N-terminal region and it is secreted through the C-terminal autotransporter of pertactin of *B*. *pertussis*. Pertactin causes pertussis in the trachea in humans. When the model of ChloroIBP was aligned to pertactin using the "super" command of PyMOL, the 331 residues of ChloroIBP aligned to the region of pertactin spanning residues 46 to 371. The resulting superposition contained 178 alpha-carbons matching with an RMSD of 0.48 Å. As expected, these matched alpha-carbons are all found within the core region of the ChloroIBP beta-solenoid structure rather than in the large loops. Therefore, it is possible that ChloroIBP is derived from a pertactin-like protein that was involved in binding to a repetitive structure on a cell surface (glycoproteins, polysaccharides) that has some similarity to ice.

There is little evidence for HGT as an explanation for the appearance of ChloroIBP in microalgae. There are too few isolates of this protein to develop a hypothesis. The freezing environment acts as a strong evolutionary pressure to select IBPs from functionally different gene products or to spread IBP genes by HGT. It is well worth surveying other microorganisms for the presence of ChloroIBP or its homologues to investigate the HGT hypothesis or provide an alternative hypothesis to explain the origin of this novel IBP.

Gene regulation of *IBPs* has been studied in Antarctic sea ice diatoms. Some isoforms of *F*. *cylindrus IBPs* were up-regulated by elevated salt concentration and subzero temperature even though strong up-regulation of the *F*. *cylindrus IBP* was not observed at subzero temperatures alone [57]. Expression of *IBP* from *Chaetoceros neogracile* (*Cn-AFP*) was investigated as a function of temperature [58]. The transcript levels from *Cn-AFP* increased by greater than 1.5-fold compared to normal transcription of the gene following gradual freezing of the culture media. As reported here for *Chloromonas* sp., the IBP gene was constantly expressed under ordinary culture condition at 3°C, and again gene expression was moderately increased under freezing conditions.

Ice-binding activity of microalgal IBPs can be detected in culture media by observing deformation of ice surfaces [14,16,22,59]. Recently, intracellular production of the AFP from *Chaetoceros neogracile* was reported [60]. Gwak and his colleagues (2014) proposed that the placement of *C*. *neogracile* AFP (Cn-AFP) near the chloroplasts prevented growth of intracellular ice crystals and helped maintain photosynthesis in the Antarctic marine diatom. Here *Chloromonas* sp. was shown to secrete its IBP into the culture media. *Chloromonas* sp. living in freshwater, such as transitory ponds, might increase gene expression of *IBP* and secret the IBP into the extracellular region under freezing conditions, which could generate narrow spaces of small volume near the microorganisms without spreading the IBP too far away.

The mature ChloroIBP is a Cys-rich protein in which 14 of its 15 Cys residues are predicted to form disulfide crosslinkages. Disulfide bonds play an important role in stabilizing proteins in the oxidizing extracellular environment. There are several other examples of highly disulfide bonded IBPs such as the 14-kDa lectin-like type II AFP in fishes, which has five disulfide bonds where most homologues have only two or three [61], and insect AFPs like *Tm*AFP (eight disulfide bonds in a 8.4 kDa protein) [62], midge AFP (eight disulfide bonds in a 8.2 kDa protein) [63], and sbwAFP (four disulfide bonds in a 9.3 kDa protein) [64]. It is quite likely that disulfide bonding helps ensure the stability of these protein folds at low temperatures where the hydrophobic effect is weakened [17]. It is interesting to note that all seven predicted disulfide bridges in ChloroIBP are grouped on one side of the beta-solenoid, the side from which the loops protrude. In some cases they appear to constrain the loop, although the details of loop topology could not be resolved by modeling. Typically the bonded cysteines are nearest neighbours or one removed. This is normal for beta-solenoid structures where the three-dimensional fold arises linearly from the addition of coils to the solenoid.

The ice-binding sites (IBS) of the hyperactive antifreeze proteins from spruce budworm (*Choristoneura fumiferana*) and mealworm (*Tenebrio molitor*), have repetitions of TXT (where X is any amino acid pointing into the structure) motifs on one of the β-helical sides [65–67]. A wider repetitive TXT motif (TXTXTXT) can be found in the AFP of longhorn beetle (*Rhagium inquisitor*), and its relative *Rhagium mordax* [68–70]. It has been suggested that IBSs with regular TXT motifs on neighbouring β-strands aligned along a flat β-sheet can organize ice-like waters that merge with, and freeze to the quasi-liquid layer above the ice lattice [71]. Based on the topological alignment of the ChloroIBP amino acid sequence with 1DAB as a template protein, it is proposed that ChloroIBP also has a β-solenoidal structure [41]. This is a right-handed solenoid with a left-handed twist. What is exciting about this model is that five T-X-T motifs and one T-X-W all line up in a regular array on one of the two flat beta-sheet faces (the β-2 face) towards the C-terminal end. Thus, we predict this is the IBS of ChloroIBP.

The strategy used to investigate the importance of these motifs was to substitute Thr residues of the ice-binding surface with Tyr and then observe the changes in antifreeze and ice-binding activities. These kinds of mutagenic studies have been carried out with AFPs originating from bacteria, fungi, yeast, insects, and plants [18,19,28,29,37]. Single or double steric mutants of several AFPs were reported to show reduced or completely abolished antifreeze activity compared to that of wild-type AFPs. The significant loss of activity in all three mutants where a single Thr on the β2 face of ChloroIBP was replaced by Tyr provided strong evidence that this is the IBS.

As a further check of the β-solenoidal structure of ChloroIBP two hydrophobic core mutations were made. Both of these completely abolished thermal hysteresis, presumably because the protein fold was destabilized.

## Materials and Methods

### Culture conditions and identification of microalgal strain

*Chloromonas* sp. was provided from Dr. Sung-Ho Kang at Korea Polar Research Institute (KOPRI). This strain was collected from transitory pools near the shore by the King Sejong Station, the Korean Antarctic station (62° 13’ S, 58° 47’ W). No specific permission was required for these locations and activities because the area near the King Sejong Station was not restricted to investigate any microorganisms. In addition, we confirm that the field studies did not involve endangered or protected species. This strain was maintained in Bolds Basal (BB) medium at 4°C (±0.5°C) under continuous light with white fluorescence at a photon flux density of 25 μmol photons m^-2^s^-1^.

To identify *Chloromonas* sp., 18S rDNA (small subunit, SSU) and internal transcribed spacer (ITS) sequences were analyzed. Genomic DNA was manually extracted by the method of Steiner *et al*. with slight modifications [72]. Nuclear SSU sequences were amplified with two pairs of primers [73]. SSU1, the leading part of SSU, was amplified with the forward primer G01 (5’-CACCTGGTTGATCTGCCAG-3’) and the reverse one G14 (5’-CCTTGGCAGACGCTTTC GCAG-3’). Sequences of SSU2, the trailing part of SSU, were amplified using forward primer G04 (5’-CAGAGGTGAAATTCTTGGAT-3’) and reverse primer G07 (5’-GCTT GATCCTTCTGCAGGTTCACCTAC-3’). These two parts of SSU overlapped to produce a complete SSU sequence. Sequences of ITS (ITS1-5.8S rDNA-ITS2) were amplified using a forward primer (ITS1, 5’-TCCGTAGGTGAACCTGCGG-3’) and a reverse primer (ITS4, 5’-GCTGCGTTCTTCATCGATGC-3’). Sequences of the two nuclear DNAs were compared to the Genbank database by the Blastn algorithm. To verify relationships with other microalgal strains, the sequences were aligned by the ClustalW method in the BioEdit program [74] and a phylogenetic analysis using maximum parsimony (MP), maximum likelihood (ML), and distance methods was performed by the program MEGA6 [25].

### Isolation of the open-reading frame encoding an ice-binding protein of *Chloromonas* sp.

Total RNA was extracted from the microalgae by the Trizol (Invitrogen, Carlsbad, CA, USA) method, according to the manufacturer’s protocol. The four sets of primers used to elucidate sequences of the *Chloromonas* sp. IBP (S1 Table) were designed from IBP mRNA sequences of *Chlamydomonas* sp. CCMP681 [22] and *Chlorella vulgaris* NJ-7 [75]. To isolate the open-reading frame (ORF) for ChloroIBP, we synthesized cDNA from 1 μg of total RNA of *Chloromonas* sp. using an oligo (dT)_18_ primer and Superscript III reverse transcriptase (Invitrogen). Four polymerase chain reactions were performed using genomic and complementary DNA as templates. The sizes of amplified products were checked on 0.8% and 1.2% agarose gels with ethidium bromide staining for genomic and cDNA amplicons, respectively. DNA bands predicted to be related to IBPs were extracted using a gel extraction kit. Purified samples of DNA were cloned using a TA cloning kit (Macrogen, Korea) and transformed into competent HIT-DH5α *E*.*coli* cells (RBC, Taiwan). After transformation, clones were inoculated into LB broth with 100 μg/ml ampicillin, and plasmid DNAs were purified using a plasmid extraction kit. Plasmids containing insert DNA were confirmed by PCR and DNA sequencing with M13-pUC primers. The isolated nucleotide sequences of the ORFs of *Chloromonas* sp. IBP (ChloroIBP) were compared to the database of NCBI by tBlastx algorithm. The deduced amino acid sequences of ChloroIBP were investigated to find the signal peptides and determine the subcellular localization by the programs of SignalP v4.0 [23] and TargetP v1.1 [76]. N-glycosylation as a post-translational modification of native ChloroIBP was predicted by NetGlyc program in the ExPASy package [27]. The mapping of introns and exons in genomic DNA sequences of ChloroIBP was manually performed by alignment with sequences of genomic DNA and ORFs of ChloroIBP.

### Phylogenetic analysis of IBPs and AFPs from *Chloromonas* sp. and other organisms showing antifreeze activity

The deduced amino acid sequence of ChloroIBP was compared to those of other IBPs and antifreeze proteins (AFPs) produced by bacteria, fungi, microalgae, and plants. The amino acid sequences for this phylogenetic analysis were collected from the NCBI database (*Glaciozyma antarcticum* AFP, ACX31168; *Lentinula edodes* IBP, ACL27146; *Typhula ishikariensis* AFP, BAD02897; *Leucosporidium* sp. AFP, ACU30807; *Fragilariopsis cylindrus* IAP, ACX36851; *Fragilariopsis curta* IAP, ACT99642; *Navicula glaciei* IBP, AAZ76254; *Chaetoceros neogracile* AFP, ACU09498; *Marinomonas primoryensis* AFP, ABL74378; *Chlamydomonas* sp. IBP-1, ABY64758; *Chlamydomonas* sp. IBP-2, ABY64759; *Chlamydomonas* sp. IBP-3, ABY64760; *Chlamydomonas* sp. IBP-4, ABY64761; *Secale cereale* IBP, AAG53609; *Daucus carota* IBP, AAC62932; *Deschampsia antarctica* IBP, ACN38296; *Lolium perenne* IRIP, ACG63781). The peptide sequences of IBPs and AFPs from the organisms were aligned using a ClustalW program in the packages of the BioEdit program [74]. The phylogenetic analyses were performed by the 5,000 bootstrap replications of distance method from the MEGA6 program [25].

### Investigation of the gene family of ChloroIBP

Southern blotting to find the number of ChloroIBP genes in the gene family was performed as previously described [58]. Genomic DNA (10 μg) extracted using the method of Fulton *et al*. [77] was digested with restriction endonucleases (*Bam*HI, *Kpn*I, and *Xba*I) and then separated on a 0.7% agarose gel. DNA fragments separated by electrophoresis were transferred to a nylon membrane (HybondTM-N+, Amersham). ChloroIBP DNA labeled by incorporation with α^32^P-dCTP (Table 1) was used as a probe following hybridization protocols used for northern blot analysis (Stratagene, USA).

### Physiological characterization of ChloroIBP

To analyze the transcriptional and translational fluctuation of IBP expression in *Chloromonas* sp. (ChloroIBP), northern and western blotting experiments were performed. For analyzing the effect of freezing conditions on transcription, algae were cultured in 25, 50 or 100% ice slush. To examine how thermal stresses affected ChloroIBP expression levels, samples were incubated for 30 min, 1 h, and 2 h at 25°C under continuous light. Northern blot analysis was done as previously described [58].

To analyze ChloroIBP production levels, samples were prepared under the same conditions as those used for northern blot analysis. Western blotting was performed as previously described [58] using antisera raised in rabbits to the CESYTENQITGNTWTG peptide sequence of ChloroIBP (residues 306 to 321, inclusive in Fig 4).

### Localization of native ChloroIBP

To confirm the subcellular localization of native ChloroIBP, microalgal strains and culture media were separated by centrifugation. For collection of intracellular proteins, harvested cells were washed three times with a buffer (20 mM Tris-HCl, pH 7.4) and lysed by vortexing for 2 min. After centrifugation at 4°C, clear supernatants and pellets were used as soluble intracellular proteins and cell debris, respectively. The culture medium filtered through a 0.2 μm membrane filter (Millipore) was used as the extracellular fraction. Extracellular proteins were concentrated 20 times for analysis due to the relatively low concentrations. Cellular location of ChloroIBP was revealed by western blot analysis.

### Biochemical characteristics of native ChloroIBP

The change of in-gel mobility of ChloroIBP by the reducing reagent β-mercaptoethanol (Sigma) was analyzed by immunoblotting with a ChloroIBP-specific polyclonal antibody. After treatment with β-mercaptoethanol, reduced proteins were alkylated by iodoacetamide (Sigma) to prevent reversible disulfide bonding [78]. The mobility of native ChloroIBP was analyzed by native polyacrylamide gel electrophoresis and western blot analysis. The 12% native polyacrylamide gel was prepared without sodium dodecyl sulfate. Samples were loaded on the gel at the concentrations of 37.5 and 75.0 μg/ml. Protein markers, BSA (bovine serum albumin, Promega) and LDH (L-lactic dehydrogenase from rabbit muscle, Sigma) were used to indicate the relative mobility of two different sized proteins of 66 and 140 kDa, respectively. PVDF membrane, onto which native extracellular proteins were transferred, was immersed in Ponceau solutions (Sigma) to mark the locations of 66 and 140 kDa reference proteins. The localization and topological changes in native ChloroIBP by reducing reagent were detected by western blot analysis [58]. Glycosylation of native ChloroIBP was detected by the use of a Pierce Glycoprotein Staining Kit (Thermo Scientific, USA).

### Expression, purification, and site-directed mutagenesis of recombinant ChloroIBP

The DNA sequences encoding the mature ChloroIBP were obtained by PCR using a forward primer with a *Bam*HI recognition site and reverse primer with an *Xho*I site. The PCR products were purified and cloned into a TA cloning vector (Macrogen, Korea). After extraction of plasmid DNA, insertion of mature ChloroIBP was confirmed by PCR using M13-pUC primers and the plasmids were sequenced to check for any mutation. The corresponding plasmids and pET-32a (+) expression vector (Novagen, USA) were digested by *Bam*HI and *Xho*I, and then the digested products were extracted from 0.8% agarose gels. The expression vector and the insert DNA of mature IBP were ligated and transformed into DH5α competent cells. The plasmids were purified, and then verified by digestion with *Bam*HI. The expression plasmids, designated Trx-ChloroIBP, were transformed into BL21 (DE3) pLysS competent cells to suppress basal expression. A single colony was inoculated into Luria-Bertani (LB) media (10 ml) containing ampicillin (100μg/ml) and incubated at 37°C with overnight shaking. An aliquot (5 ml) of the culture was transferred into 500 ml of LB media and incubated at 37°C with continuous shaking until the OD_600_ reached 0.4. Isopropyl β-D-thiogalactopyranoside (IPTG) was added to the culture to a final concentration of 1 mM and then shaking incubation was performed for 3 h. After induction of protein, cultured cells were harvested by centrifugation (19,000 x g for 20 min at 4°C) and resuspended in a lysis buffer (20 mMTris-HCl, pH 8.8 and 25 mM PMSF). The cells were lysed by sonication for 3 min on icy water (5-sec pulse and 8-sec delay) and centrifuged (19,000 x g, 20 min, 4°C) to collect supernatants as soluble fractions. Whole lysate and soluble fractions of ChloroIBP were analyzed on 12% SDS-PAGE. The purification of recombinant ChloroIBP (Trx-ChloroIBP) was performed by affinity chromatography on Ni-NTA resin (Elpisbio, Korea). Each of the fractions (500 μL) of elution buffer (0.25 M NaCl, 20 mMTris-HCl, pH 8.0, 0.5 M imidazole) was collected and analyzed by 12% SDS-PAGE. The heterologous expression construct was prepared by ligation of pET-32a(+) and the coding regions of mature ChloroIBP, with the 23-residue signal peptide removed.

Site-directed mutagenesis was performed by a PCR-based process with mutagenic primer pairs and Trx-ChloroIBP plasmid as the template DNA. After verification of the nucleotide sequences of mutated plasmids, mutant Trx-ChloroIBPs were over-expressed as described for the recombinant Trx-ChloroIBP.

### Measurement of thermal hysteresis

The thermal hysteresis (TH) activity and ice crystal morphology of Trx-ChloroIBP were observed by using a nanolitre-osmometer (Otago Nanolitre-osmometer, New Zealand) linked to an optical microscope (BX-53, Olympus, Japan). Each hole in the sample discs was loaded with protein samples and positioned on a temperature-controlled stage. The samples were frozen at less than -20°C and then maintained at this frozen status for 3 min. Samples at the freezing stage were warmed at the rate of 5°C min^-1^ until only one single crystal remained. The temperature at which only a single ice crystal melted was determined as the melting point. The temperature was decreased at the rate of 0.01°C every 5 sec and the freezing points were determined as the temperature at which the ice crystal growth began. BSA solution (5 mg/ml) was used as a control for TH measurements. The values of TH were designated by differences of temperature between the melting points and the freezing points. Pictures of ice crystal morphology were captured by a CCD imaging system (DMC e310 Plus, Korea).

### Protein modeling and molecular dynamics

For modeling of ChloroIBP, structural homology was first examined by the program Phyre2 [39]. Results from the intensive mode of analysis identified 1DAB (*Bordetella pertussis* virulent factor P.69 pertactin) as a suitable homology modeling guide for ChloroIBP. Homology models were produced from manually-generated sequence alignments using Modeller version 9.9 [40] and visualized using PyMol program [79]. At least 100 models were made and the best model (based on the Modeller Objective Function score) was tested by running it though a molecular dynamics simulation using version 4.5.3 of the program Gromacs [80,81]. All molecular dynamics runs used the following protocol. The molecule was placed in the center of a triclinic box of water such that the edge of the box was 0.9 nanometers away from the protein in all dimensions. The charge of the system (protein plus water) was neutralized by the addition of 14 sodium ions and the system was minimized using a maximum of 5000 steps with a force tolerance of 100 kJ/mol. The water positions were relaxed using a molecular dynamics run of 10 ps duration at a temperature of 273 K, in which the protein atoms were restrained and the waters were free to move. After that, an unrestrained NVT molecular dynamics simulation was run for 20 ns at a temperature of 273 K. Long-range electrostatics were treated with the particle mesh Ewald model using a cutoff of 1 nm and the temperature coupling used the v-rescale model.

## Supporting Information

S1 Fig Deduced amino acid sequences of ChloroIBP gene and protein. The signal peptide sequence is underlined. Possible N-glycosylation sites are indicated by upper asterisks.
(PDF)

S2 Fig Alignment of ChloroIBP amino acid sequence with four isoforms of *Chlamydomonas* sp. CCMP681 by the ClustalW algorithm [24]. Dots indicate amino acids identical to those of the ChloroIBP sequence. Dashes indicate gaps in sequence alignment.
(PDF)

S3 Fig Prediction of N-glycosylation sites of ChloroIBP by the NetGlyc program in the ExPASy package [27]. Residues 122N and 248N were predicted to be N-glycosylated by exceeding the threshold of 0.5 (0.61 for 122N and 0.59 for 248N).
(PDF)

S4 Fig Heterologous protein expression and affinity chromatography of ChloroIBP. SDS-PAGE analysis of protein fractions: M, protein marker; 1, Un-induced crude *E*. *coli* extract; 2, Total crude extract after induction by IPTG; 3, Soluble fractions after sonication method; 4, Insoluble pellet after sonication; 5, Fractions of Trx-ChloroIBP from Ni-NTA affinity chromatography.
(PDF)

S5 Fig Structural characteristics of ChloroIBP as a modeled β-solenoid protein. (A) Right-handed β-solenoid with left-handed twist. Broken and red arrows represent the axis of the β-solenoid and the direction of the solenoidal twist, respectively. Beta-strands are indicated by thick green arrows. N and C indicate the N and C termini, respectively; (B) Distribution of outward facing threonine residues (magenta) on one beta-sheet of the solenoid organized into two parallel rows. C-terminal end at the top; (C) End on view of the solenoid with hydrophobic core residues coloured orange.
(PDF)

S6 Fig The averaged CD spectra for Trx-ChloroIBP. (PDF)

S7 Fig The plots of (A) root-mean-square (RMS) deviation, (B) radius of gyration and (C) RMS fluctuation of the Trx-ChloroIBP. The root-mean-square deviation (RMSD) plot shows two lines, the black line is for the alpha carbons of the core, excluding the loops (residues 124–134, 152–163, 211–223 and 301–307), while the grey line is for the alpha carbons of just the loops.
(PDF)

S8 Fig Ice crystal morphology directed by wild-type and mutant ChloroIBPs as a function of protein concentration. Concentrations of wild-type and mutant ChloroIBPs are described at the left of the wild-type ChloroIBP. Scale bars indicate 100 μm.
(PDF)

S1 Table Primers used in this study. For indicates a forward primer; Rev is a reverse primer. Sequences underlines are restriction sites.
(PDF)

S2 Table Distribution of exons, introns, and splicing signals in the ChloroIBP gene. Numbers refer to base pairs.
(PDF)

S3 Table Secondary structure content of ChloroIBP calculated from CD spectra. The secondary structure content (α-helix, beta-strand, beta-turn and random coil) of the 373-residue ChloroIBP was determined as the difference between the secondary structure contents of the 495-residue Trx-ChloroIBP fusion protein and the 122-residue thioredoxin domain. The latter was taken directly from the crystal structure of thioredoxin fused to a splicing factor (PDB code: 3DXB) using the program Stride. CD analysis was performed as previously described [35].
(PDF)
