# Supplementary material for: New Cysteine-Rich Ice-Binding Protein Secreted from Antarctic Microalga, Chloromonas sp
Source: PLoS One. 2016 Apr 20;11(4):e0154056. doi: 10.1371/journal.pone.0154056 (PMC4838330; doi:10.1371/journal.pone.0154056)
Supplement: S1 Fig — The signal peptide sequence is underlined. Possible N-glycosylation sites are indicated by upper asterisks. (PDF) [file pone.0154056.s001.pdf]

```
ATG CCT AGC TCT TCA ATG AAG CTG TTT GCA GCA CTA CTA GTC GCC TGC CTG GCT CAG ACC    60
 M   P   S   S   S   M   K   L   F   A   A   L   L   V   A   C   L   A   Q   T

AGC ATG GCG GCC ATC GTT GTG TGC AAG ATG GAT GCC CAG GAT GGA GAC ACG CTT ACT GCC   120
 S   M   A   A   I   V   V   C   K   M   D   A   Q   D   G   D   T   L   T   A

GCC TGC AGC GTG GGT GTC TCA GGT CAA CCC ATC TCT CTT GTG GGG CCA GGA TCT GGC CAG   180
 A   C   S   V   G   V   S   G   Q   P   I   S   L   V   G   P   G   S   G   Q

CAG CAG CTG ACT GGA TCG CAA GTC TCA TAC ACC CTG GAT GTG AAT GCC AGA TCA ACA CTG   240
 Q   Q   L   T   G   S   Q   V   S   Y   T   L   D   V   N   A   R   S   T   L

TTC GAG TGC GCC AGT GAG GAT GAC CTG CTC ATC ATT GAC TCA TCC CAG TAC AGC AGC CAG   300
 F   E   C   A   S   E   D   D   L   L   I   I   D   S   S   Q   Y   S   S   Q

ACC CTC AAC AAC TGC GAA CCC CCT CTG CTT GAG TTG CGC GGG TGC AGC AAT GCC ATC CTC   360
 T   L   N   N   C   E   P   P   L   L   E   L   R   G   C   S   N   A   I   L

     *
AGC AAC AAC ACC TTC ATT AGC ATC ACA CGG AGC ACT GCC CAG CCT GGG TGC ACC ATC AGC   420
 S   N   N   T   F   I   S   I   T   R   S   T   A   Q   P   G   C   T   I   S

AAG TAC GGC CCA TGC GTG GCC GTA GTG GGC GCT GCC AGC CAA GAG ACG GAC TGG TCT TTC   480
 K   Y   G   P   C   V   A   V   V   G   A   A   S   Q   E   T   D   W   S   F

TCT TCA CTC GCC AAC ACC TTC ACC TCC ACA ATC TGC TCA TCT ATT TCA GCA ACC TCC GGC   540
 S   S   L   A   N   T   F   T   S   T   I   C   S   S   I   S   A   T   S   G

CGC CTA GGT GGT GCA TTT GCA TTC GAG CAC AAT GAC TCA CCA GGC GCC ATG TCA GCA GTC   600
 R   L   G   G   A   F   A   F   E   H   N   D   S   P   G   A   M   S   A   V

GTC AAG GGA TCT ACC TTC ACC AGC ACC GCT TGT GAC TTT GGA GGA GCC ATT CAC AGC GCC   660
 V   K   G   S   T   F   T   S   T   A   C   D   F   G   G   A   I   H   S   A

AAT GCA TCC CTT ACT CTA ACC GAC TCA ACA TTC ACG GGC ACT TTG GCA GTG GAT GGA GGC   720
 N   A   S   L   T   L   T   D   S   T   F   T   G   T   L   A   V   D   G   G

                             *
GCT GTG CAG TTC GTC GGC ACC AAT GCC ACT GTT GCT CCT ATC CAG AAG CTT CAA GTG AAG   780
 A   V   Q   F   V   G   T   N   A   T   V   A   P   I   Q   K   L   Q   V   K

TCA TCT ACC TTC ACT TCA AAC ACG GCT GTG ACC ACT GGC GGA ATC ATC CAA GTG ACA GGG   840
 S   S   T   F   T   S   N   T   A   V   T   T   G   G   I   I   Q   V   T   G

GGT GCT GTG TCC ATT GAT GGC TCA ACG TTC ACC AAC GGC GAG GCC CAG ATT GGA CAG TGT   900
 G   A   V   S   I   D   G   S   T   F   T   N   G   E   A   Q   I   G   Q   C

GTG TGG CTT GAT AAG TGC GAG AGC TAC ACC GAG AAT CAG ATC ACA GGC AAC ACC TGG ACA   960
 V   W   L   D   K   C   E   S   Y   T   E   N   Q   I   T   G   N   T   W   T

GGC TGT GCC AAA CCC GAG AGC CCC CCA ATC TCA TGG TGC AAG GCT CAT GAT GGC AAC AAT  1020
 G   C   A   K   P   E   S   P   P   I   S   W   C   K   A   H   D   G   N   N

TGG ACC ACC TGC GGA ATG GAA GGG CCC AGG GAG TGC TAC TAA                          1062
 W   T   T   C   G   M   E   G   P   R   E   C   Y   *
```
