# Supplementary material for: New Cysteine-Rich Ice-Binding Protein Secreted from Antarctic Microalga, Chloromonas sp
Source: PLoS One. 2016 Apr 20;11(4):e0154056. doi: 10.1371/journal.pone.0154056 (PMC4838330; doi:10.1371/journal.pone.0154056)
Supplement: S2 Fig — Dots indicate amino acids identical to those of the ChloroIBP sequence. Dashes indicate gaps in sequence alignment. (PDF) [file pone.0154056.s002.pdf]

```
                                                                      60
Chloro_IBP    --MPSSSMK- ---LFAALLV ACLAQTSMAA IVVCKMDAQD GDTLTAACSV GVSGQPISLV

Chlamy_IBP_1  --.......- ---......I ..M....... .......... .N........ .......P.E
Chlamy_IBP_2  --.STT.I.W ATP.L.-... .S....G..S ....T.N.I. .TI.AG.... ..N.AVV...
Chlamy_IBP_3  --.T.TDSS- ILTIL...M. VS.T.A.-.. K.T.T.N.IS .RQVGGT... -FG..VT.FI
Chlamy_IBP_4  MSATTTT..W VTP.L..... .S.T...... ........I. .QI..G..T. ....AV....

                                                                     120
Chloro_IBP    GPGSGQQQLT GSQVSYTLDV NARSTLFECA SEDDLLIIDS SQYSSQTLNN CEPPLLELRG

Chlamy_IBP_1  .....P...P .........A .N.......G D.....V... .......... ..........
Chlamy_IBP_2  ...T.P...A ..E....... .......... .......... .T..G..... ..........
Chlamy_IBP_3  .T.T.SFP.P .TDIT..I.A .G..M..... .QS.I.TV.. .V..G..I.. .....I.F..
Chlamy_IBP_4  ...A.P.L.N D.SI...... .......... .......... .......... .D........

                                                                     180
Chloro_IBP    CSNAILSNNT FISITRSTAQ P-GCTISKYG PCVAVVGAAS QETDWSFSSL ANTFTSTICS

Chlamy_IBP_1  ........T. .......... .-E....... ..M......T .......... .........A
Chlamy_IBP_2  ....VF.... .T........ .-E....... ....I..... ..S....... E......A.A
Chlamy_IBP_3  ....AVI.TK .TD....N.. .DN.EV.QF. ..I....SV. .TE..T...N ET...T..V.
Chlamy_IBP_4  .......H.. .T........ .-A....... ....I..... .VA....... D.....SG.A

                                                                     240
Chloro_IBP    SISATSG-RL GGAFAFEHND SPGAMSAVVK GSTFTSTACD FGGAIHSANA SLTLTDSTFT

Chlamy_IBP_1  .......-.V .......R.. .....T.... .......... .......... ..........
Chlamy_IBP_2  ..A....-.. ...L.LVR.G A......LI. K......M.. .........V ....QS.V..
Chlamy_IBP_3  .LQPSAVS.. ...I.VLKK. .V..VQ.... .T...T.S.. L.....T.GV ...AM.T...
Chlamy_IBP_4  ..A....-.. ...L.LVRA. .G.....FIQ K......Q.. .........V ....QS....

                                                                     300
Chloro_IBP    GTLAVDGGAV QFVGTNATVA PIQKLQVKSS TFTSNTAVTT GGIIQVTGGA VSIDGSTFTN

Chlamy_IBP_1  .......... L......... ...Q.S.... ....S..... ..M.E.A... ....D.....
Chlamy_IBP_2  S.......G. D.RAADP.AV .T.A.V.DRT ....P..... ..A...R..V .T.KD.....
Chlamy_IBP_3  TNS.....SI H.T.-----. ADAQFV.QRC ...TQK.TAN ..S.A.Y..N .T.TD.D.SE
Chlamy_IBP_4  SS........ N.IAASPLAV .T.T.L.DR. ...AP..... ..A.Q.N..L LT.KD.....

                                                                     360
Chloro_IBP    GEAQIGQCVW LDKCESYTEN QITGNTWTGC AKPESPPISW CKAHDGNNWT TCGMEGPREC

Chlamy_IBP_1  .D.K..K... .........D K..N...... .......... .......K.. ..........
Chlamy_IBP_2  ..SL..E... .AN.G....S E.V....... ..RG...... .RE....D.A .......V..
Chlamy_IBP_3  .R.TQ.A... .NQ.A...KD ..VN.K..D. STQFT---TL .CPAN..V.S S..IA..A..
Chlamy_IBP_4  .A.LN.K... V.I.QT...T E.VR...... .T..G..... .......Q.. S.......Y.

                         371
Chloro_IBP    Y--------- -

Chlamy_IBP_1  .--------- -
Chlamy_IBP_2  .--------- -
Chlamy_IBP_3  SEAVPPALCP A
Chlamy_IBP_4  .--------- -
```
