# Supplementary material for: New Cysteine-Rich Ice-Binding Protein Secreted from Antarctic Microalga, Chloromonas sp
Source: PLoS One. 2016 Apr 20;11(4):e0154056. doi: 10.1371/journal.pone.0154056 (PMC4838330; doi:10.1371/journal.pone.0154056)

```
MPSSSMKLFAALLVACLAQTSMAAIVVCKMDAQDGDTLTAACSVGVSGQPISLVGPGSGQQQLTGSQVSYTLDVNARSTL      80
FECASEDDLLIIDSSQYSSQTLNNCEPPLLELRGCSNAILSNNTFISITRSTAQPGCTISKYGPCVAVVGAASQETDWSF     160
SSLANTFTSTICSSISATSGRLGGAFAFEHNDSPGAMSAVVKGSTFTSTACDFGGAIHSANASLTLTDSTFTGTLAVDGG     240
AVQFVGTNATVAPIQKLQVKSSTFTSNTAVTTGGIIQVTGGAVSIDGSTFTNGEAQIGQCVWLDKCESYTENQITGNTWT     320
GCAKPESPPISWCKAHDGNNWTTCGMEGPRECY
................................................................................      80
.........................................N......................................     160
................................................................................     240
.......N........................................................................     320
.................................                                                    400
```

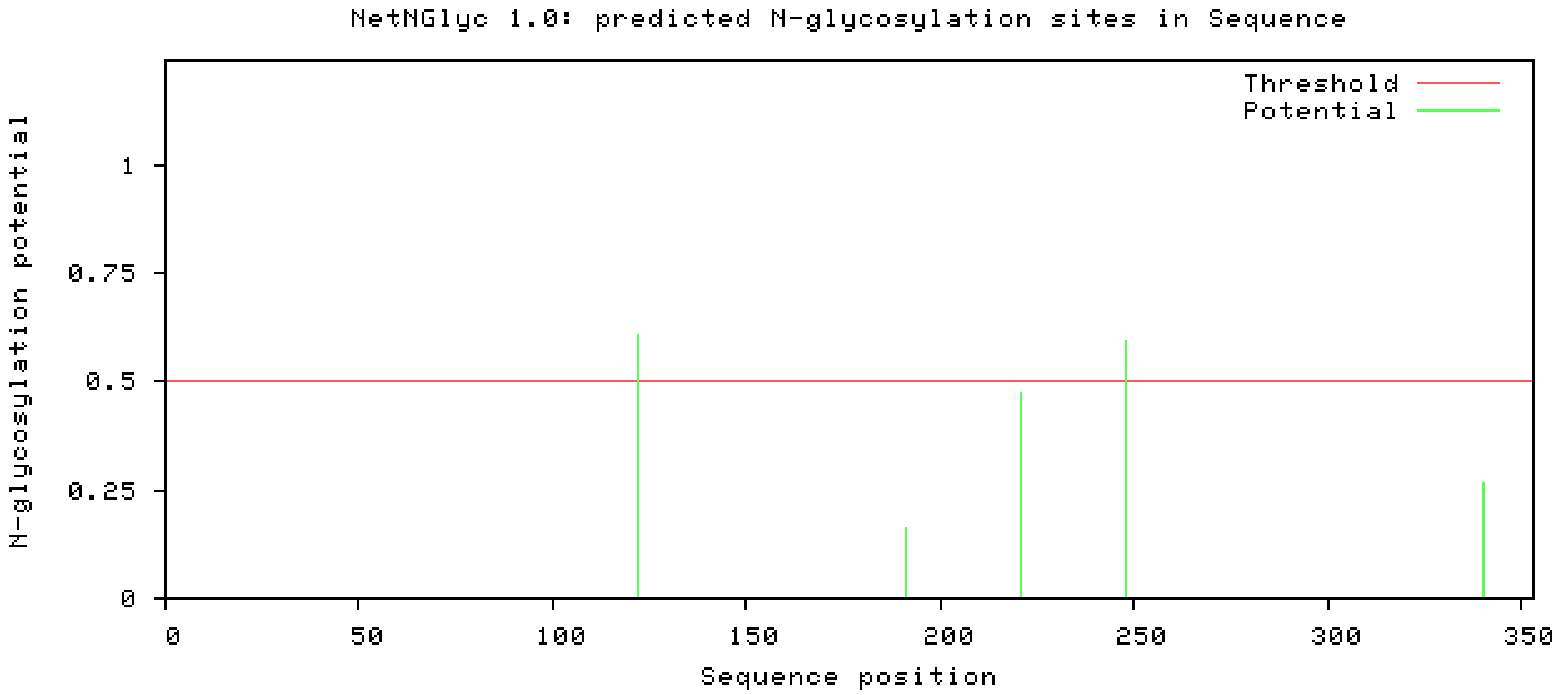

Supplement: S3 Fig — Residues 122N and 248N were predicted to be N-glycosylated by exceeding the threshold of 0.5 (0.61 for 122N and 0.59 for 248N). (PDF) [file pone.0154056.s003.pdf]
