# Supplementary figures and images for: New Cysteine-Rich Ice-Binding Protein Secreted from Antarctic Microalga, Chloromonas sp
Source: PLoS One. 2016 Apr 20;11(4):e0154056. doi: 10.1371/journal.pone.0154056 (PMC4838330; doi:10.1371/journal.pone.0154056)

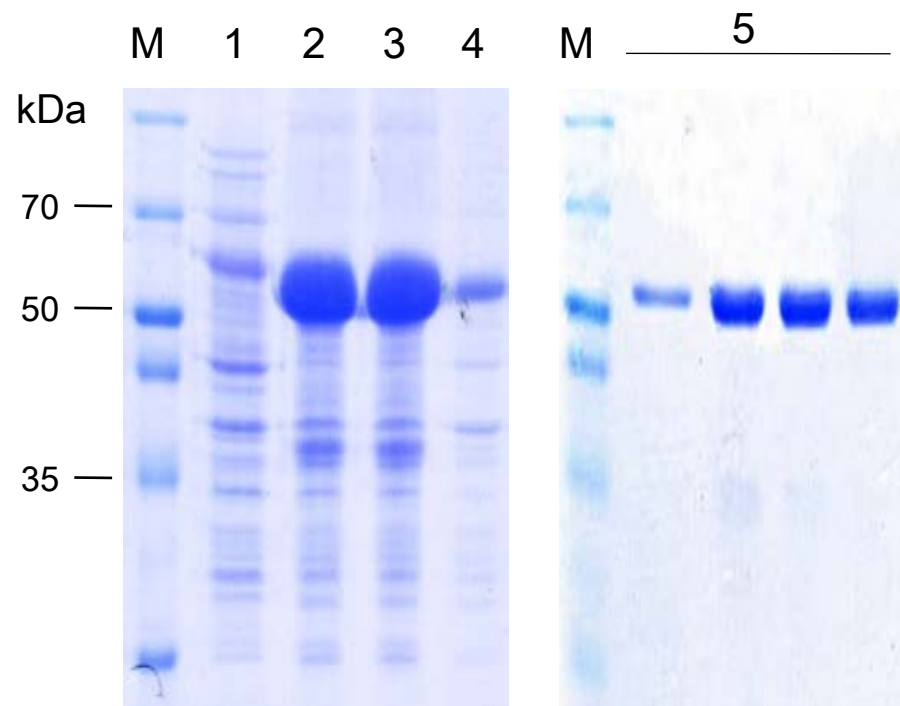

Supplement: S4 Fig — SDS-PAGE analysis of protein fractions: M, protein marker; 1, Un-induced crude E. coli extract; 2, Total crude extract after induction by IPTG; 3, Soluble fractions after sonication method; 4, Insoluble pellet after sonication; 5, Fractions of Trx-ChloroIBP from Ni-NTA affinity chromatography. (PDF) [file pone.0154056.s004.pdf]

(A)

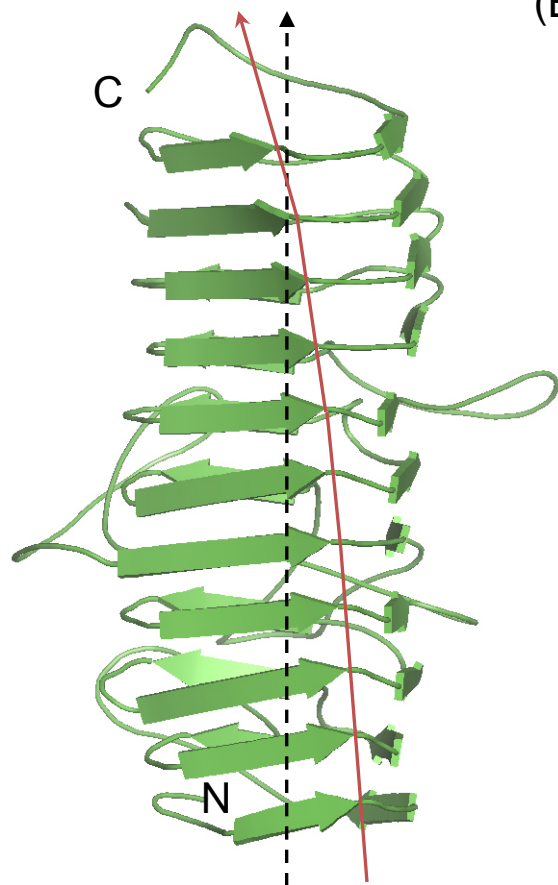

(B)

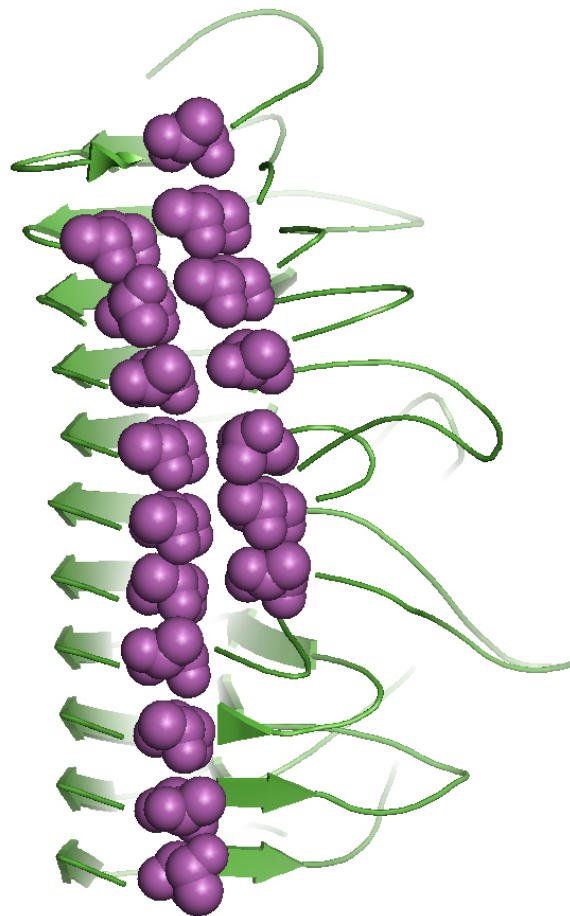

(C)

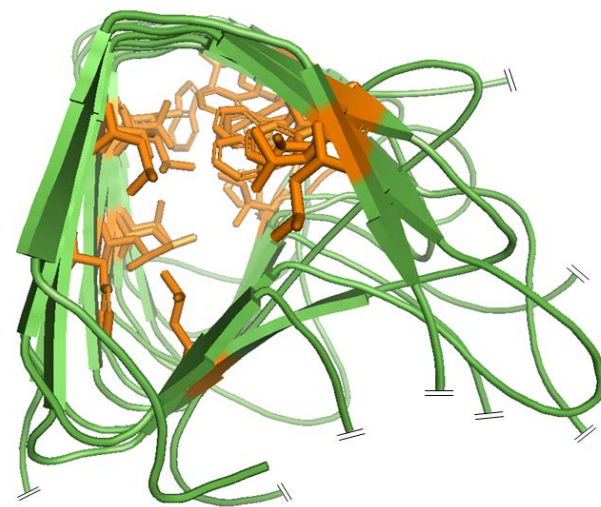

Supplement: S5 Fig — (A) Right-handed β-solenoid with left-handed twist. Broken and red arrows represent the axis of the β-solenoid and the direction of the solenoidal twist, respectively. Beta-strands are indicated by thick green arrows. N and C indicate the N and C termini, respectively; (B) Distribution of outward facing threonine residues (magenta) on one beta-sheet of the solenoid organized into two parallel rows. C-terminal end at the top; (C) End on view of the solenoid with hydrophobic core residues coloured orange. (PDF) [file pone.0154056.s005.pdf]

**CD Spectrum of Chloro IBP (including Trx Tag)  
at 25 °C**

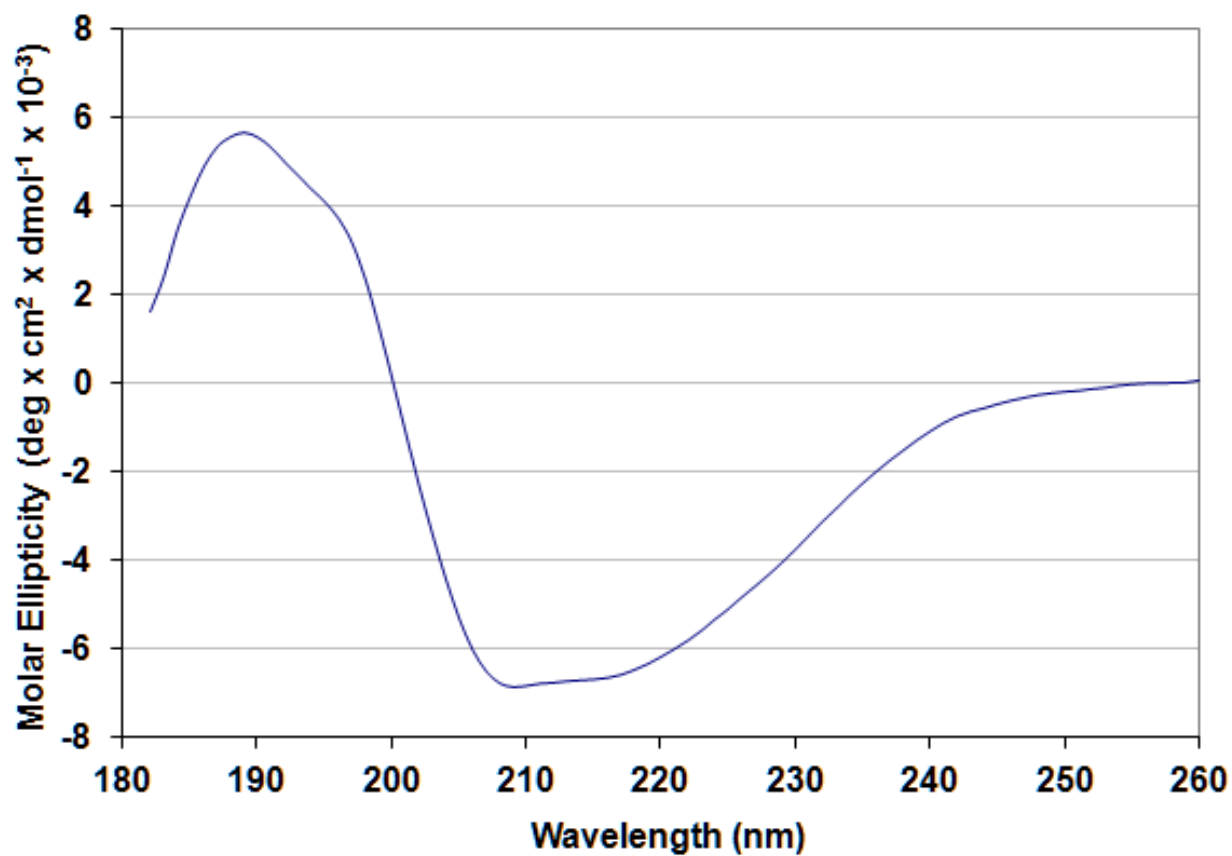

Supplement: S6 Fig — (PDF) [file pone.0154056.s006.pdf]

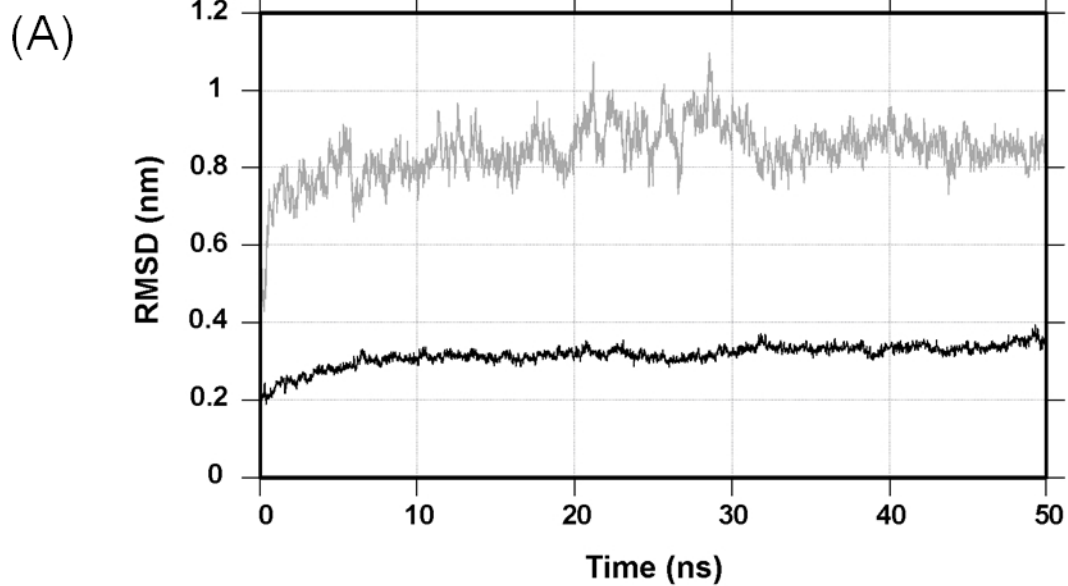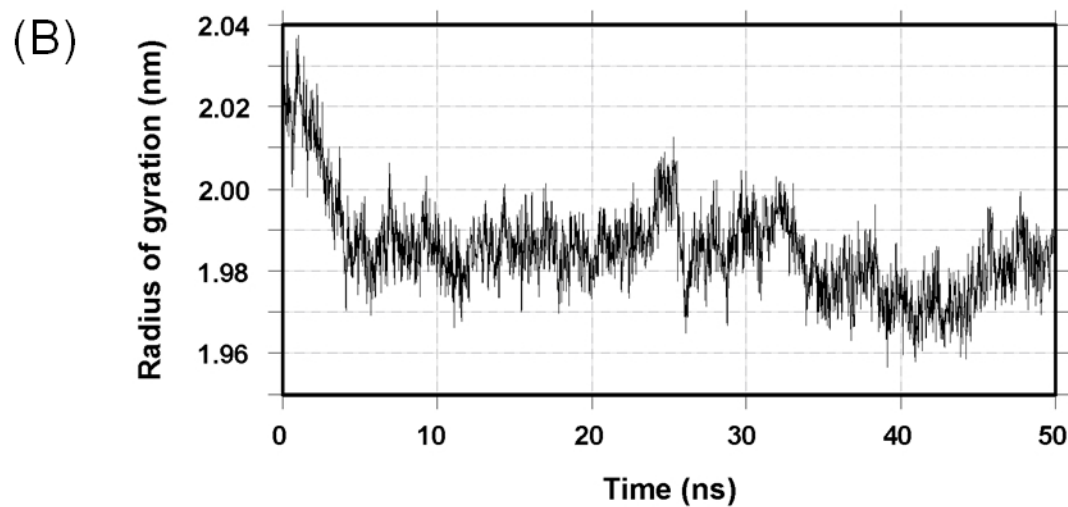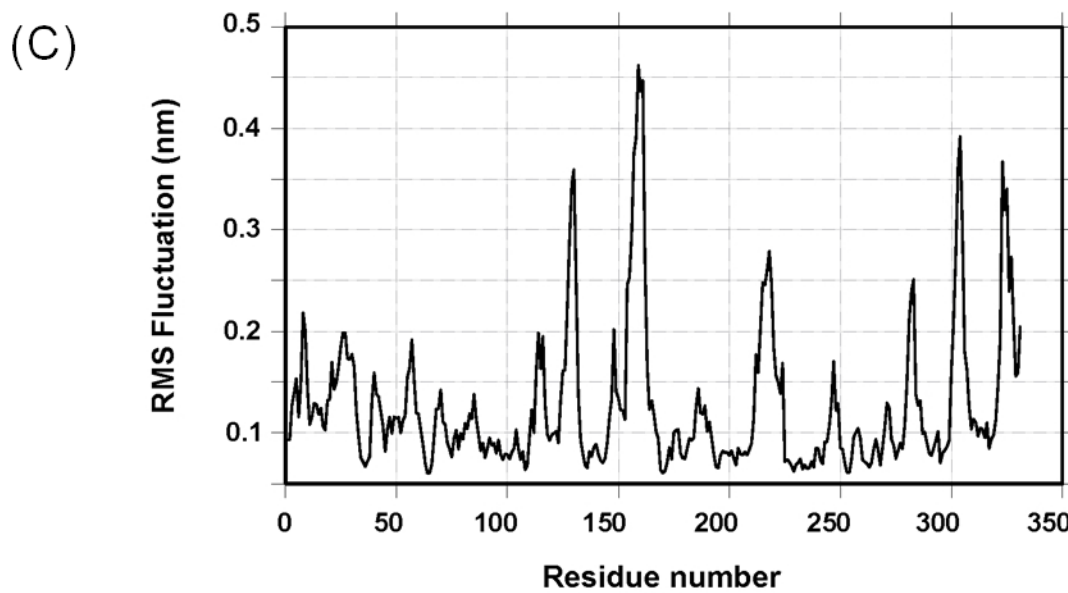

Supplement: S7 Fig — The root-mean-square deviation (RMSD) plot shows two lines, the black line is for the alpha carbons of the core, excluding the loops (residues 124–134, 152–163, 211–223 and 301–307), while the grey line is for the alpha carbons of just the loops. (PDF) [file pone.0154056.s007.pdf]
