# Supplementary material for: New Cysteine-Rich Ice-Binding Protein Secreted from Antarctic Microalga, Chloromonas sp
Source: PLoS One. 2016 Apr 20;11(4):e0154056. doi: 10.1371/journal.pone.0154056 (PMC4838330; doi:10.1371/journal.pone.0154056)
Supplement: S8 Fig — Concentrations of wild-type and mutant ChloroIBPs are described at the left of the wild-type ChloroIBP. Scale bars indicate 100 μm. (PDF) [file pone.0154056.s008.pdf]

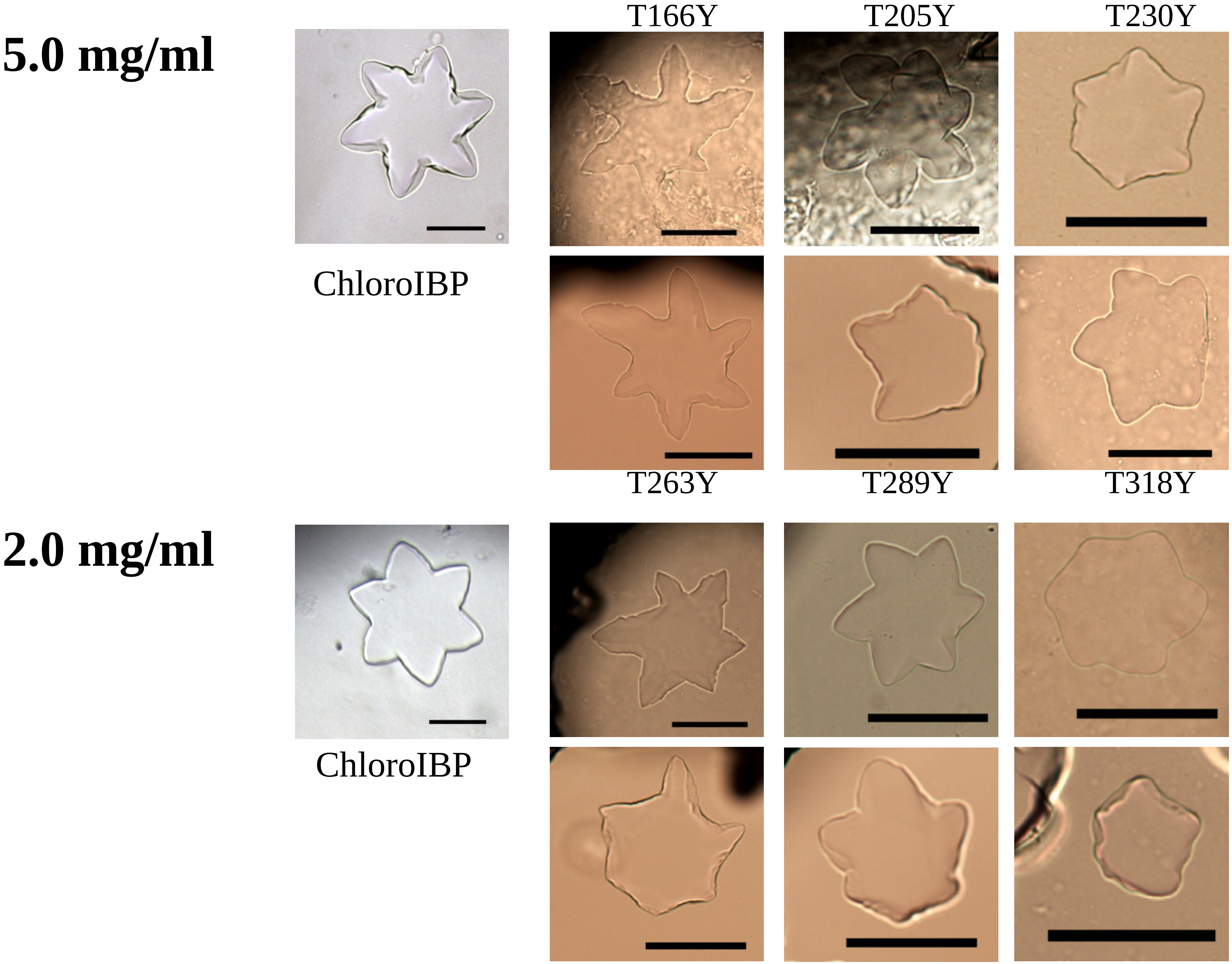
5.0 mg/ml
T166Y
T205Y
T230Y
ChloroIBP
T263Y
T289Y
T318Y
2.0 mg/ml
ChloroIBP

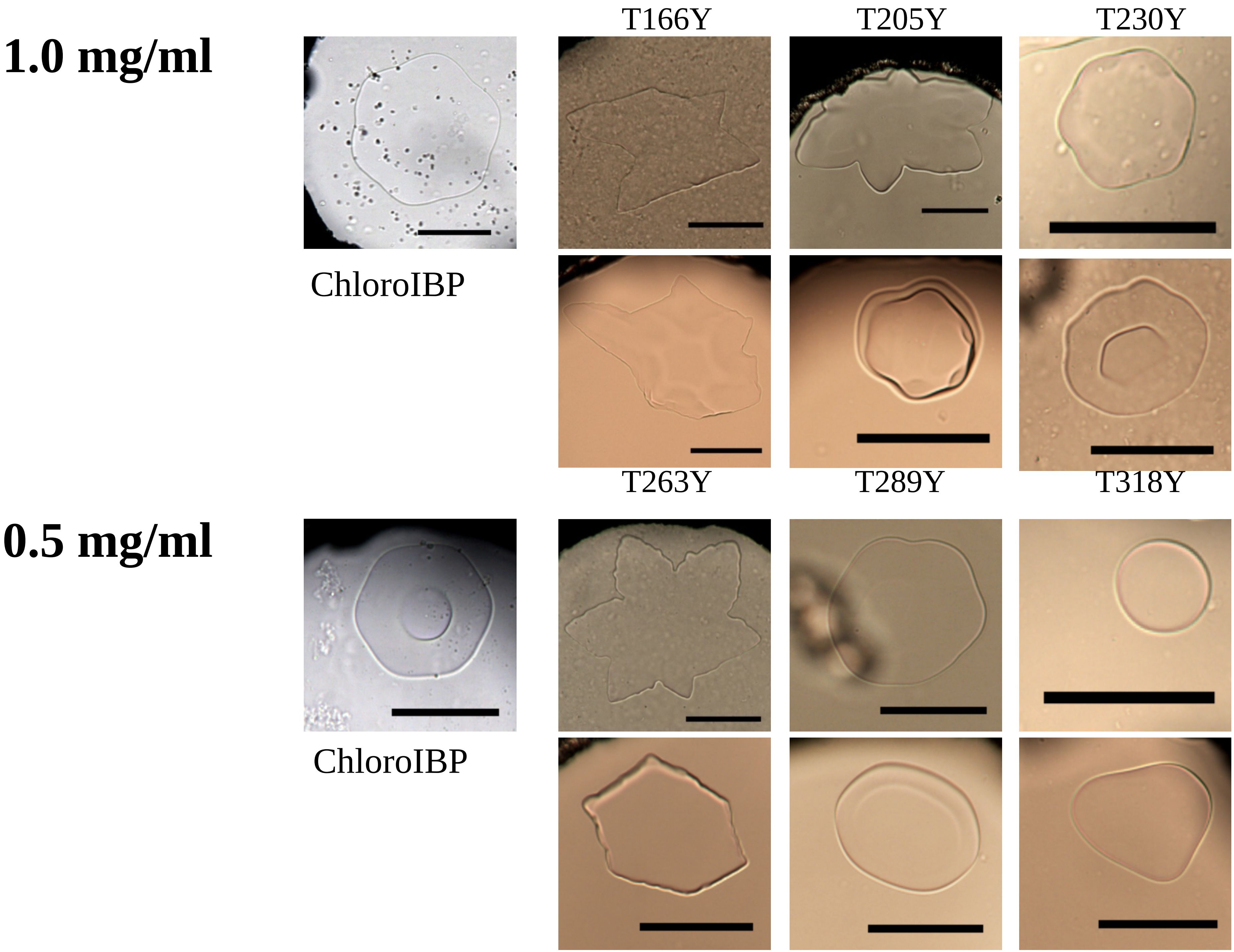
1.0 mg/ml
T166Y
T205Y
T230Y
ChloroIBP
T263Y
T289Y
T318Y
0.5 mg/ml
ChloroIBP

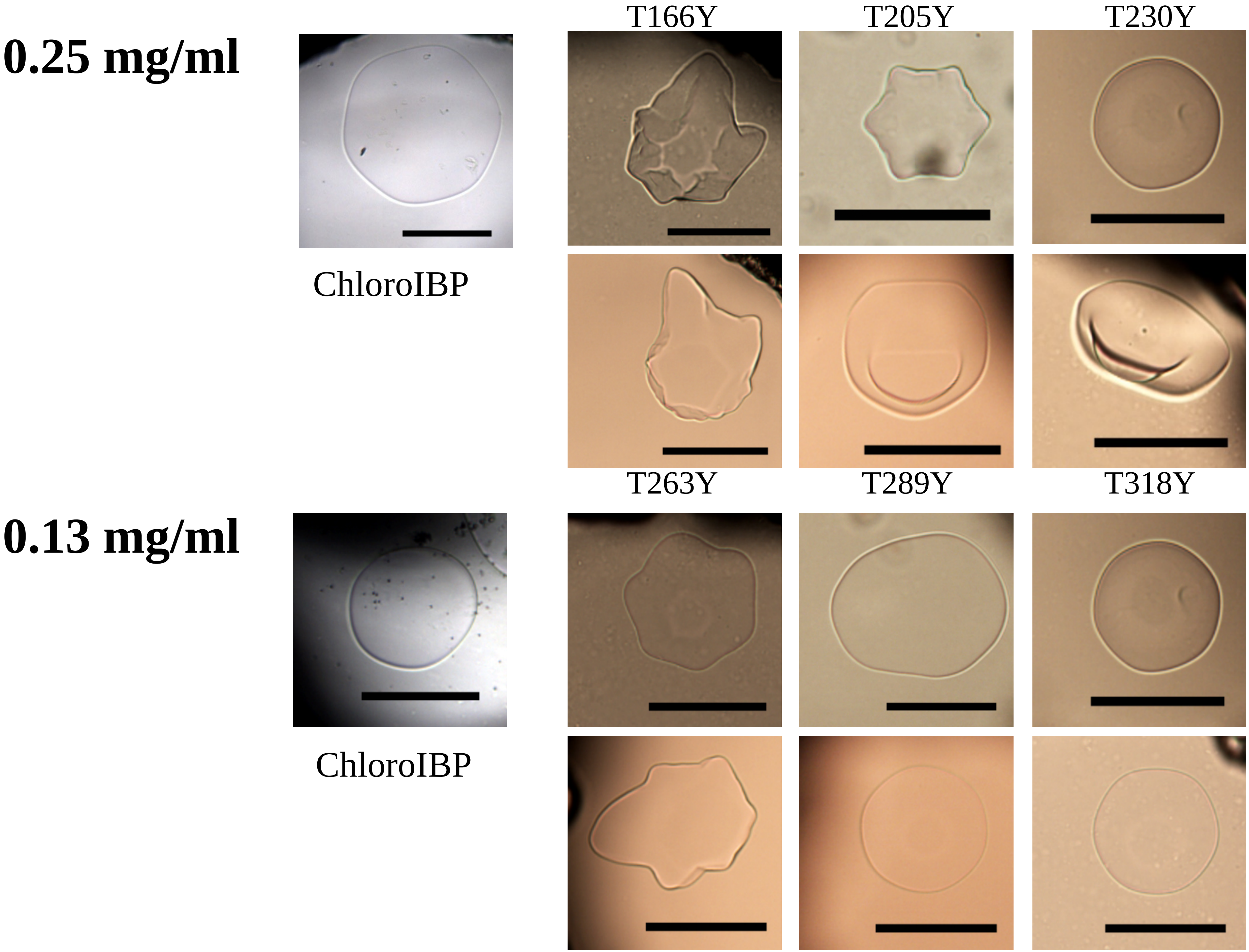
0.25 mg/ml
T166Y
T205Y
T230Y
ChloroIBP
T263Y
T289Y
T318Y
0.13 mg/ml
ChloroIBP

5.0 mg/ml

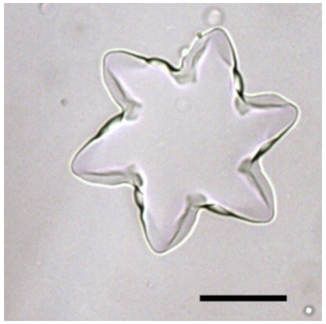

ChloroIBP

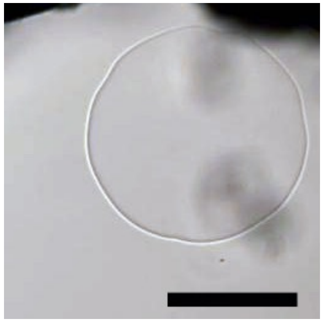

F160S

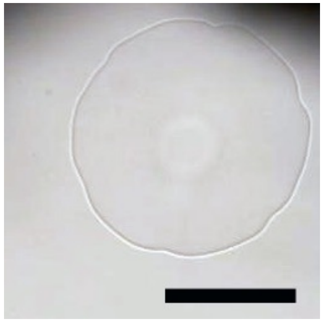

L226S
