# Supplementary material for: New Cysteine-Rich Ice-Binding Protein Secreted from Antarctic Microalga, Chloromonas sp
Source: PLoS One. 2016 Apr 20;11(4):e0154056. doi: 10.1371/journal.pone.0154056 (PMC4838330; doi:10.1371/journal.pone.0154056)
Supplement: S1 Table — For indicates a forward primer; Rev is a reverse primer. Sequences underlines are restriction sites. (PDF) [file pone.0154056.s009.pdf]

Name of primers	Sequences of primers (5'→3')
**IBP of green microalgae**	
CCMP681_1_For	ATGCCTAGCTCTTCAATGAAGC
CCMP681_1_Rev	TTAGTAGCACTCCCTGGGCC
CCMP681_2_For	ATGTCTACCACGAGCATAAAGT
CCMP681_2_Rev	TTAGTAACACTCGACTGGCC
CCMP681_3_For	ATGTCAGCAACAACCACCAC
CCMP681_3_Rev	TTAATAGAATAACGAGGCCCCT
C_vul_For	ATGCAGGCCGCAGCCATGTC
C_vul_Rev	TTAGAGCTTGTTAGCCTCATTC
**Inner primers to elucidate DNA sequences of gDNA**	
gDNA_inner_For	CCTAGCAACAACACCTTCA
gDNA_inner_Rev	AAGCCACACACACTGTCCAA
**Probe for Southern blot**	
Southern_For	GGCTCGAATCCAAGCATTTA
Southern_Rev	CATGGTCGCATGCTATTGTC
**Probe for Northern blot**	
Northern_For	GTTGTGTGAAGATGGATGC
Northern_Rev	CGGAGGTTGTGAAATAGA
**Expression of Chloromonas IBP**	
Chloromoas_IBP_For	CGCC<u>GGATCC</u>ATGGCCATCGTTGTGTGCAAGAT
Chloromoas_IBP_Rev	GCGG<u>CTCGAG</u>TTAGTAGCACTCCCTGGGCC
