# Supplementary material for: New Cysteine-Rich Ice-Binding Protein Secreted from Antarctic Microalga, Chloromonas sp
Source: PLoS One. 2016 Apr 20;11(4):e0154056. doi: 10.1371/journal.pone.0154056 (PMC4838330; doi:10.1371/journal.pone.0154056)
Supplement: S2 Table — Numbers refer to base pairs. (PDF) [file pone.0154056.s010.pdf]

Number of exons	Start and stop points of exons in genomic DNA		Length of exons	Number of introns	Length of introns	Splicing * signals
	Start	Stop				
E01	1	128	128			
				I01	126	GT/AG
E02	255	343	89			
				I02	117	GT/AG
E03	461	576	116			
				I03	217	GT/AG
E04	794	979	186			
				I04	210	GT/AG
E05	1190	1278	89			
				I05	215	GT/AG
E06	1494	1581	88			
				I06	192	GT/AG
E07	1174	1821	48			
				I07	198	GT/AG
E08	2020	2079	60			
				I08	167	GT/AG
E09	2247	2281	35			
				I09	123	GT/AG
E10	2405	2482	78			
				I10	158	GT/AG
E11	2641	2766	126			
				I11	436	GT/AG
E12	3203	3221	19			

* Canonical splicing signal : GT/AG
