# Supplementary material for: New Cysteine-Rich Ice-Binding Protein Secreted from Antarctic Microalga, Chloromonas sp
Source: PLoS One. 2016 Apr 20;11(4):e0154056. doi: 10.1371/journal.pone.0154056 (PMC4838330; doi:10.1371/journal.pone.0154056)
Supplement: S3 Table — The secondary structure content (α-helix, beta-strand, beta-turn and random coil) of the 373-residue ChloroIBP was determined as the difference between the secondary structure contents of the 495-residue Trx-ChloroIBP fusion protein and the 122-residue thioredoxin domain. The latter was taken directly from the crystal structure of thioredoxin fused to a splicing factor (PDB code: 3DXB) using the program Stride. CD analysis was performed as previously described [35]. (PDF) [file pone.0154056.s011.pdf]

Structural elements	Total<br>with TRX (%)	TRX (%)	Total<br>without TRX (%)
α-helix	13	31	7
Beta-strand	34	23	38
Beta-turn	20	19	20
Random coil	33	27	35
Total	100	100	100
